# Refining the diagnosis of gestational diabetes mellitus: a systematic review and meta-analysis

**DOI:** 10.1038/s43856-023-00393-8

**Published:** 2023-12-18

**Authors:** Ellen C. Francis, Camille E. Powe, William L. Lowe, Sara L. White, Denise M. Scholtens, Jiaxi Yang, Yeyi Zhu, Cuilin Zhang, Marie-France Hivert, Soo Heon Kwak, Arianne Sweeting, ADA/EASD PMDI, Deirdre K. Tobias, Jordi Merino, Abrar Ahmad, Catherine Aiken, Jamie L. Benham, Dhanasekaran Bodhini, Amy L. Clark, Kevin Colclough, Rosa Corcoy, Sara J. Cromer, Daisy Duan, Jamie L. Felton, Pieter Gillard, Véronique Gingras, Romy Gaillard, Eram Haider, Alice Hughes, Jennifer M. Ikle, Laura M. Jacobsen, Anna R. Kahkoska, Jarno L. T. Kettunen, Raymond J. Kreienkamp, Lee-Ling Lim, Jonna M. E. Männistö, Robert Massey, Niamh-Maire Mclennan, Rachel G. Miller, Mario Luca Morieri, Jasper Most, Rochelle N. Naylor, Bige Ozkan, Kashyap Amratlal Patel, Scott J. Pilla, Katsiaryna Prystupa, Sridharan Raghavan, Mary R. Rooney, Martin Schön, Zhila Semnani-Azad, Magdalena Sevilla-Gonzalez, Pernille Svalastoga, Wubet Worku Takele, Claudia Ha-ting Tam, Anne Cathrine B. Thuesen, Mustafa Tosur, Amelia S. Wallace, Caroline C. Wang, Jessie J. Wong, Jennifer M. Yamamoto, Katherine Young, Chloé Amouyal, Mette K. Andersen, Maxine P. Bonham, Mingling Chen, Feifei Cheng, Tinashe Chikowore, Sian C. Chivers, Christoffer Clemmensen, Dana Dabelea, Adem Y. Dawed, Aaron J. Deutsch, Laura T. Dickens, Linda A. DiMeglio, Monika Dudenhöffer-Pfeifer, Carmella Evans-Molina, María Mercè Fernández-Balsells, Hugo Fitipaldi, Stephanie L. Fitzpatrick, Stephen E. Gitelman, Mark O. Goodarzi, Jessica A. Grieger, Marta Guasch-Ferré, Nahal Habibi, Torben Hansen, Chuiguo Huang, Arianna Harris-Kawano, Heba M. Ismail, Benjamin Hoag, Randi K. Johnson, Angus G. Jones, Robert W. Koivula, Aaron Leong, Gloria K. W. Leung, Ingrid M. Libman, Kai Liu, S. Alice Long, Robert W. Morton, Ayesha A. Motala, Suna Onengut-Gumuscu, James S. Pankow, Maleesa Pathirana, Sofia Pazmino, Dianna Perez, John R. Petrie, Camille E. Powe, Alejandra Quinteros, Rashmi Jain, Debashree Ray, Mathias Ried-Larsen, Zeb Saeed, Vanessa Santhakumar, Sarah Kanbour, Sudipa Sarkar, Gabriela S. F. Monaco, Elizabeth Selvin, Wayne Huey-Herng Sheu, Cate Speake, Maggie A. Stanislawski, Nele Steenackers, Andrea K. Steck, Norbert Stefan, Julie Støy, Rachael Taylor, Sok Cin Tye, Gebresilasea Gendisha Ukke, Marzhan Urazbayeva, Bart Van der Schueren, Camille Vatier, John M. Wentworth, Wesley Hannah, Sara L. White, Gechang Yu, Yingchai Zhang, Shao J. Zhou, Jacques Beltrand, Michel Polak, Ingvild Aukrust, Elisa de Franco, Sarah E. Flanagan, Kristin A. Maloney, Andrew McGovern, Janne Molnes, Mariam Nakabuye, Pål Rasmus Njølstad, Hugo Pomares-Millan, Michele Provenzano, Cécile Saint-Martin, Cuilin Zhang, Yeyi Zhu, Sungyoung Auh, Russell de Souza, Andrea J. Fawcett, Chandra Gruber, Eskedar Getie Mekonnen, Emily Mixter, Diana Sherifali, Robert H. Eckel, John J. Nolan, Louis H. Philipson, Rebecca J. Brown, Liana K. Billings, Kristen Boyle, Tina Costacou, John M. Dennis, Jose C. Florez, Anna L. Gloyn, Maria F. Gomez, Peter A. Gottlieb, Siri Atma W. Greeley, Kurt Griffin, Andrew T. Hattersley, Irl B. Hirsch, Marie-France Hivert, Korey K. Hood, Jami L. Josefson, Lori M. Laffel, Siew S. Lim, Ruth J. F. Loos, Ronald C. W. Ma, Chantal Mathieu, Nestoras Mathioudakis, James B. Meigs, Shivani Misra, Viswanathan Mohan, Rinki Murphy, Richard Oram, Katharine R. Owen, Susan E. Ozanne, Ewan R. Pearson, Wei Perng, Toni I. Pollin, Rodica Pop-Busui, Richard E. Pratley, Leanne M. Redman, Maria J. Redondo, Rebecca M. Reynolds, Robert K. Semple, Jennifer L. Sherr, Emily K. Sims, Arianne Sweeting, Tiinamaija Tuomi, Miriam S. Udler, Kimberly K. Vesco, Tina Vilsbøll, Robert Wagner, Stephen S. Rich, Paul W. Franks

**Affiliations:** 1 Department of Biostatistics and Epidemiology, Rutgers School of Public Health, Piscataway, NJ USA; 2 Diabetes Unit, Massachusetts General Hospital, Boston, MA USA; 3 Department of Medicine, Northwestern University Feinberg School of Medicine, Chicago, IL USA; 4 Department of Women and Children’s Health, King’s College London, London, UK; 5 Department of Preventive Medicine, Division of Biostatistics, Northwestern University Feinberg School of Medicine, Chicago, IL USA; 6 Global Center for Asian Women’s Health (GloW), Yong Loo Lin School of Medicine, National University of Singapore, Singapore, Singapore; 7 Department of Obstetrics and Gynecology, Yong Loo Lin School of Medicine, National University of Singapore, Singapore, Singapore; 8 Bia-Echo Asia Centre for Reproductive Longevity & Equality (ACRLE), Yong Loo Lin School of Medicine, National University of Singapore, Singapore, Singapore; 9 Kaiser Permanente Northern California Division of Research, Oakland, CA USA; 10 Department of Nutrition, Harvard T.H. Chan School of Public Health, Boston, MA USA; 11 Department of Population Medicine, Harvard Medical School, Harvard Pilgrim Health Care Institute, Boston, MA USA; 12 Department of Internal Medicine, Seoul National University College of Medicine, Seoul National University Hospital, Seoul, Republic of Korea; 13 Faculty of Medicine and Health, University of Sydney, Sydney, NSW Australia; 14 Division of Preventative Medicine, Department of Medicine, Brigham and Women’s Hospital and Harvard Medical School, Boston, MA USA; 15 Novo Nordisk Foundation Center for Basic Metabolic Research, Faculty of Health and Medical Sciences, University of Copenhagen, Copenhagen, Denmark; 16 Diabetes Unit, Endocrine Division, Massachusetts General Hospital, Boston, MA USA; 17 Center for Genomic Medicine, Massachusetts General Hospital, Boston, MA USA; 18 Department of Clinical Sciences, Lund University Diabetes Centre, Lund University Malmö, Sweden; 19 Department of Obstetrics and Gynaecology, the Rosie Hospital, Cambridge, UK; 20 NIHR Cambridge Biomedical Research Centre, University of Cambridge, Cambridge, UK; 21 Departments of Medicine and Community Health Sciences, Cumming School of Medicine, University of Calgary, Calgary, AB Canada; 22 Department of Molecular Genetics, Madras Diabetes Research Foundation, Chennai, India; 23 Division of Pediatric Endocrinology, Department of Pediatrics, Saint Louis University School of Medicine, SSM Health Cardinal Glennon Children’s Hospital, St. Louis, MO USA; 24 Department of Clinical and Biomedical Sciences, University of Exeter Medical School, Exeter, Devon UK; 25 CIBER-BBN, ISCIII, Madrid, Spain; 26 Institut d’Investigació Biomèdica Sant Pau (IIB SANT PAU), Barcelona, Spain; 27 Departament de Medicina, Universitat Autònoma de Barcelona, Bellaterra, Spain; 28 Programs in Metabolism and Medical & Population Genetics, Broad Institute, Cambridge, MA USA; 29 Department of Medicine, Harvard Medical School, Boston, MA USA; 30 Division of Endocrinology, Diabetes and Metabolism, Johns Hopkins University School of Medicine, Baltimore, MD USA; 31 Department of Pediatrics, Indiana University School of Medicine, Indianapolis, IN USA; 32 Herman B Wells Center for Pediatric Research, Indiana University School of Medicine, Indianapolis, IN USA; 33 Center for Diabetes and Metabolic Diseases, Indiana University School of Medicine, Indianapolis, IN USA; 34 University Hospital Leuven, Leuven, Belgium; 35 Department of Nutrition, Université de Montréal, Montreal, QC Canada; 36 Research Center, Sainte-Justine University Hospital Center, Montreal, QC Canada; 37 Department of Pediatrics, Erasmus Medical Center, Rotterdam, The Netherlands; 38 Division of Population Health & Genomics, School of Medicine, University of Dundee, Dundee, UK; 39 Department of Pediatrics, Stanford School of Medicine, Stanford University, Stanford, CA USA; 40 Stanford Diabetes Research Center, Stanford School of Medicine, Stanford University, Stanford, CA USA; 41 University of Florida, Gainesville, FL USA; 42 Department of Nutrition, University of North Carolina at Chapel Hill, Chapel Hill, NC USA; 43 Helsinki University Hospital, Abdominal Centre/Endocrinology, Helsinki, Finland; 44 Folkhalsan Research Center, Helsinki, Finland; 45 Institute for Molecular Medicine Finland FIMM, University of Helsinki, Helsinki, Finland; 46 Department of Pediatrics, Division of Endocrinology, Boston Children’s Hospital, Boston, MA USA; 47 Department of Medicine, Faculty of Medicine, University of Malaya, Kuala Lumpur, Malaysia; 48 Asia Diabetes Foundation, Hong Kong SAR, China; 49 Department of Medicine & Therapeutics, Chinese University of Hong Kong, Hong Kong SAR, China; 50 Departments of Pediatrics and Clinical Genetics, Kuopio University Hospital, Kuopio, Finland; 51 Department of Medicine, University of Eastern Finland, Kuopio, Finland; 52 Centre for Cardiovascular Science, Queen’s Medical Research Institute, University of Edinburgh, Edinburgh, UK; 53 Department of Epidemiology, University of Pittsburgh, Pittsburgh, PA USA; 54 Metabolic Disease Unit, University Hospital of Padova, Padova, Italy; 55 Department of Medicine, University of Padova, Padova, Italy; 56 Department of Orthopedics, Zuyderland Medical Center, Sittard-Geleen, The Netherlands; 57 Departments of Pediatrics and Medicine, University of Chicago, Chicago, IL USA; 58 Welch Center for Prevention, Epidemiology, and Clinical Research, Johns Hopkins Bloomberg School of Public Health, Baltimore, MD USA; 59 Ciccarone Center for the Prevention of Cardiovascular Disease, Johns Hopkins School of Medicine, Baltimore, MD USA; 60 Department of Medicine, Johns Hopkins University, Baltimore, MD USA; 61 Department of Health Policy and Management, Johns Hopkins University Bloomberg School of Public Health, Baltimore, MD USA; 62 Institute for Clinical Diabetology, German Diabetes Center, Leibniz Center for Diabetes Research at Heinrich Heine University Düsseldorf, Auf’m Hennekamp 65, 40225 Düsseldorf, Germany; 63 German Center for Diabetes Research (DZD), Ingolstädter Landstraße 1, 85764 Neuherberg, Germany; 64 Section of Academic Primary Care, US Department of Veterans Affairs Eastern Colorado Health Care System, Aurora, CO USA; 65 Department of Medicine, University of Colorado School of Medicine, Aurora, CO USA; 66 Department of Epidemiology, Johns Hopkins Bloomberg School of Public Health, Baltimore, MD USA; 67 Institute of Experimental Endocrinology, Biomedical Research Center, Slovak Academy of Sciences, Bratislava, Slovakia; 68 Clinical and Translational Epidemiology Unit, Massachusetts General Hospital, Boston, MA USA; 69 Mohn Center for Diabetes Precision Medicine, Department of Clinical Science, University of Bergen, Bergen, Norway; 70 Children and Youth Clinic, Haukeland University Hospital, Bergen, Norway; 71 Eastern Health Clinical School, Monash University, Melbourne, VIC Australia; 72 Laboratory for Molecular Epidemiology in Diabetes, Li Ka Shing Institute of Health Sciences, The Chinese University of Hong Kong, Hong Kong, China; 73 Hong Kong Institute of Diabetes and Obesity, The Chinese University of Hong Kong, Hong Kong, China; 74 Department of Pediatrics, Baylor College of Medicine, Houston, TX USA; 75 Division of Pediatric Diabetes and Endocrinology, Texas Children’s Hospital, Houston, TX USA; 76 Children’s Nutrition Research Center, USDA/ARS, Houston, TX USA; 77 Stanford University School of Medicine, Stanford, CA USA; 78 Internal Medicine, University of Manitoba, Winnipeg, MB Canada; 79 Department of Diabetology, APHP, Paris, France; 80 Sorbonne Université, INSERM, NutriOmic Team, Paris, France; 81 Department of Nutrition, Dietetics and Food, Monash University, Melbourne, VIC Australia; 82 Monash Centre for Health Research and Implementation, Monash University, Clayton, VIC Australia; 83 Health Management Center, The Second Affiliated Hospital of Chongqing Medical University, Chongqing Medical University, Chongqing, China; 84 MRC/Wits Developmental Pathways for Health Research Unit, Department of Paediatrics, Faculty of Health Sciences, University of the Witwatersrand, Johannesburg, South Africa; 85 Channing Division of Network Medicine, Brigham and Women’s Hospital, Boston, MA USA; 86 Sydney Brenner Institute for Molecular Bioscience, Faculty of Health Sciences, University of the Witwatersrand, Johannesburg, South Africa; 87 Lifecourse Epidemiology of Adiposity and Diabetes (LEAD) Center, University of Colorado Anschutz Medical Campus, Aurora, CO USA; 88 Section of Adult and Pediatric Endocrinology, Diabetes and Metabolism, Kovler Diabetes Center, University of Chicago, Chicago, IL USA; 89 Department of Pediatrics, Riley Hospital for Children, Indiana University School of Medicine, Indianapolis, IN USA; 90 Richard L. Roudebush VAMC, Indianapolis, IN USA; 91 Biomedical Research Institute Girona, IdIBGi, Girona, Spain; 92 Diabetes, Endocrinology and Nutrition Unit Girona, University Hospital Dr Josep Trueta, Girona, Spain; 93 Institute of Health System Science, Feinstein Institutes for Medical Research, Northwell Health, Manhasset, NY USA; 94 University of California at San Francisco, Department of Pediatrics, Diabetes Center, San Francisco, CA USA; 95 Division of Endocrinology, Diabetes and Metabolism, Cedars-Sinai Medical Center, Los Angeles, CA USA; 96 Department of Medicine, Cedars-Sinai Medical Center, Los Angeles, CA USA; 97 Adelaide Medical School, Faculty of Health and Medical Sciences, The University of Adelaide, Adelaide, SA Australia; 98 Robinson Research Institute, The University of Adelaide, Adelaide, SA Australia; 99 Department of Public Health and Novo Nordisk Foundation Center for Basic Metabolic Research, Faculty of Health and Medical Sciences, University of Copenhagen, 1014 Copenhagen, Denmark; 100 Division of Endocrinology and Diabetes, Department of Pediatrics, Sanford Children’s Hospital, Sioux Falls, SD USA; 101 University of South Dakota School of Medicine, E Clark St, Vermillion, SD USA; 102 Department of Biomedical Informatics, University of Colorado Anschutz Medical Campus, Aurora, CO USA; 103 Department of Epidemiology, Colorado School of Public Health, Aurora, CO USA; 104 Royal Devon University Healthcare NHS Foundation Trust, Exeter, UK; 105 Oxford Centre for Diabetes, Endocrinology and Metabolism, University of Oxford, Oxford, UK; 106 Division of General Internal Medicine, Massachusetts General Hospital, Boston, MA USA; 107 UPMC Children’s Hospital of Pittsburgh, Pittsburgh, PA USA; 108 Center for Translational Immunology, Benaroya Research Institute, Seattle, WA USA; 109 Department of Pathology & Molecular Medicine, McMaster University, Hamilton, ON Canada; 110 Population Health Research Institute, Hamilton, ON Canada; 111 Department of Translational Medicine, Medical Science, Novo Nordisk Foundation, Tuborg Havnevej 19, 2900 Hellerup, Denmark; 112 Department of Diabetes and Endocrinology, Nelson R Mandela School of Medicine, University of KwaZulu-Natal, Durban, South Africa; 113 Center for Public Health Genomics, Department of Public Health Sciences, University of Virginia, Charlottesville, VA USA; 114 Division of Epidemiology and Community Health, School of Public Health, University of Minnesota, Minneapolis, MN USA; 115 Department of Chronic Diseases and Metabolism, Clinical and Experimental Endocrinology, KU Leuven, Leuven, Belgium; 116 School of Health and Wellbeing, College of Medical, Veterinary and Life Sciences, University of Glasgow, Glasgow, UK; 117 Department of Obstetrics, Gynecology, and Reproductive Biology, Massachusetts General Hospital and Harvard Medical School, Boston, MA USA; 118 Sanford Children’s Specialty Clinic, Sioux Falls, SD USA; 119 Department of Pediatrics, Sanford School of Medicine, University of South Dakota, Sioux Falls, SD USA; 120 Department of Biostatistics, Johns Hopkins Bloomberg School of Public Health, Baltimore, MD USA; 121 Centre for Physical Activity Research, Rigshospitalet, Copenhagen, Denmark; 122 Institute for Sports and Clinical Biomechanics, University of Southern Denmark, Odense, Denmark; 123 Department of Medicine, Division of Endocrinology, Diabetes and Metabolism, Indiana University School of Medicine, Indianapolis, IN USA; 124 AMAN Hospital, Doha, Qatar; 125 Institute of Molecular and Genomic Medicine, National Health Research Institutes, Taipei City, Taiwan, ROC; 126 Division of Endocrinology and Metabolism, Taichung Veterans General Hospital, Taichung, Taiwan, ROC; 127 Division of Endocrinology and Metabolism, Taipei Veterans General Hospital, Taipei, Taiwan, ROC; 128 Center for Interventional Immunology, Benaroya Research Institute, Seattle, WA USA; 129 Barbara Davis Center for Diabetes, University of Colorado Anschutz Medical Campus, Aurora, CO USA; 130 University Hospital of Tübingen, Tübingen, Germany; 131 Institute of Diabetes Research and Metabolic Diseases (IDM), Helmholtz Center Munich, Neuherberg, Germany; 132 Steno Diabetes Center Aarhus, Aarhus University Hospital, Aarhus, Denmark; 133 University of Newcastle, Newcastle upon Tyne, UK; 134 Sections on Genetics and Epidemiology, Joslin Diabetes Center, Harvard Medical School, Boston, MA USA; 135 Department of Clinical Pharmacy and Pharmacology, University Medical Center Groningen, Groningen, The Netherlands; 136 Department of Gastroenterology, Baylor College of Medicine, Houston, TX USA; 137 Department of Endocrinology, University Hospitals Leuven, Leuven, Belgium; 138 Sorbonne University, Inserm U938, Saint-Antoine Research Centre, Institute of Cardiometabolism and Nutrition, Paris, 75012 France; 139 Department of Endocrinology, Diabetology and Reproductive Endocrinology, Assistance Publique-Hôpitaux de Paris, Saint-Antoine University Hospital, National Reference Center for Rare Diseases of Insulin Secretion and Insulin Sensitivity (PRISIS), Paris, France; 140 Royal Melbourne Hospital Department of Diabetes and Endocrinology, Parkville, VIC Australia; 141 Walter and Eliza Hall Institute, Parkville, VIC Australia; 142 University of Melbourne Department of Medicine, Parkville, VIC Australia; 143 Deakin University, Melbourne, VIC Australia; 144 Department of Epidemiology, Madras Diabetes Research Foundation, Chennai, India; 145 Department of Diabetes and Endocrinology, Guy’s and St Thomas’ Hospitals NHS Foundation Trust, London, UK; 146 School of Agriculture, Food and Wine, University of Adelaide, Adelaide, SA Australia; 147 Institut Cochin, Inserm U 10116, Paris, France; 148 Pediatric Endocrinology and Diabetes, Hopital Necker Enfants Malades, APHP Centre, université de Paris, Paris, France; 149 Department of Medical Genetics, Haukeland University Hospital, Bergen, Norway; 150 Department of Medicine, University of Maryland School of Medicine, Baltimore, MD USA; 151 Department of Epidemiology, Geisel School of Medicine at Dartmouth, Hanover, NH USA; 152 Nephrology, Dialysis and Renal Transplant Unit, IRCCS—Azienda Ospedaliero-Universitaria di Bologna, Alma Mater Studiorum University of Bologna, Bologna, Italy; 153 Department of Medical Genetics, AP-HP Pitié-Salpêtrière Hospital, Sorbonne University, Paris, France; 154 Global Center for Asian Women’s Health, Yong Loo Lin School of Medicine, National University of Singapore, Singapore, Singapore; 155 Department of Epidemiology and Biostatistics, University of California San Francisco, San Francisco, CA USA; 156 National Institute of Diabetes and Digestive and Kidney Diseases, National Institutes of Health, Bethesda, MD USA; 157 Department of Health Research Methods, Evidence, and Impact, Faculty of Health Sciences, McMaster University, Hamilton, ON Canada; 158 Ann & Robert H. Lurie Children’s Hospital of Chicago, Department of Pediatrics, Northwestern University Feinberg School of Medicine, Chicago, IL USA; 159 Department of Clinical and Organizational Development, Chicago, IL USA; 160 American Diabetes Association, Arlington, VA USA; 161 College of Medicine and Health Sciences, University of Gondar, Gondar, Ethiopia; 162 Global Health Institute, Faculty of Medicine and Health Sciences, University of Antwerp, 2160 Antwerp, Belgium; 163 Department of Medicine and Kovler Diabetes Center, University of Chicago, Chicago, IL USA; 164 School of Nursing, Faculty of Health Sciences, McMaster University, Hamilton, ON Canada; 165 Division of Endocrinology, Metabolism, Diabetes, University of Colorado, Boulder, CO USA; 166 Department of Clinical Medicine, School of Medicine, Trinity College Dublin, Dublin, Ireland; 167 Department of Endocrinology, Wexford General Hospital, Wexford, Ireland; 168 Division of Endocrinology, NorthShore University HealthSystem, Skokie, IL USA; 169 Department of Medicine, Prtizker School of Medicine, University of Chicago, Chicago, IL USA; 170 Department of Genetics, Stanford School of Medicine, Stanford University, Stanford, CA USA; 171 Faculty of Health, Aarhus University, Aarhus, Denmark; 172 Departments of Pediatrics and Medicine and Kovler Diabetes Center, University of Chicago, Chicago, IL USA; 173 Sanford Research, Sioux Falls, SD USA; 174 University of Washington School of Medicine, Seattle, WA USA; 175 Department of Medicine, Universite de Sherbrooke, Sherbrooke, QC Canada; 176 Joslin Diabetes Center, Harvard Medical School, Boston, MA USA; 177 Charles Bronfman Institute for Personalized Medicine, Icahn School of Medicine at Mount Sinai, New York, NY USA; 178 Broad Institute, Cambridge, MA USA; 179 Division of Metabolism, Digestion and Reproduction, Imperial College London, London, UK; 180 Department of Diabetes & Endocrinology, Imperial College Healthcare NHS Trust, London, UK; 181 Department of Diabetology, Madras Diabetes Research Foundation & Dr. Mohan’s Diabetes Specialities Centre, Chennai, India; 182 Department of Medicine, Faculty of Medicine and Health Sciences, University of Auckland, Auckland, New Zealand; 183 Auckland Diabetes Centre, Te Whatu Ora Health New Zealand, Auckland, New Zealand; 184 Medical Bariatric Service, Te Whatu Ora Counties, Health New Zealand, Auckland, New Zealand; 185 Oxford NIHR Biomedical Research Centre, University of Oxford, Oxford, UK; 186 University of Cambridge, Metabolic Research Laboratories and MRC Metabolic Diseases Unit, Wellcome-MRC Institute of Metabolic Science, Cambridge, UK; 187 Department of Epidemiology & Public Health, University of Maryland School of Medicine, Baltimore, MD USA; 188 Department of Internal Medicine, Division of Metabolism, Endocrinology and Diabetes, University of Michigan, Ann Arbor, MI USA; 189 AdventHealth Translational Research Institute, Orlando, FL USA; 190 Pennington Biomedical Research Center, Baton Rouge, LA USA; 191 MRC Human Genetics Unit, Institute of Genetics and Cancer, University of Edinburgh, Edinburgh, UK; 192 Yale School of Medicine, New Haven, CT USA; 193 Department of Endocrinology, Royal Prince Alfred Hospital, Sydney, NSW Australia; 194 Kaiser Permanente Northwest, Kaiser Permanente Center for Health Research, Portland, OR USA; 195 Clinial Research, Steno Diabetes Center Copenhagen, Herlev, Denmark; 196 Department of Clinical Medicine, Faculty of Health and Medical Sciences, University of Copenhagen, Copenhagen, Denmark; 197 Department of Endocrinology and Diabetology, University Hospital Düsseldorf, Heinrich Heine University Düsseldorf, Moorenstr. 5, 40225 Düsseldorf, Germany

**Keywords:** Gestational diabetes, Diagnostic markers

## Abstract

**Background:**

Perinatal outcomes vary for women with gestational diabetes mellitus (GDM). The precise factors beyond glycemic status that may refine GDM diagnosis remain unclear. We conducted a systematic review and meta-analysis of potential precision markers for GDM.

**Methods:**

Systematic literature searches were performed in PubMed and EMBASE from inception to March 2022 for studies comparing perinatal outcomes among women with GDM. We searched for precision markers in the following categories: maternal anthropometrics, clinical/sociocultural factors, non-glycemic biochemical markers, genetics/genomics or other -omics, and fetal biometry. We conducted post-hoc meta-analyses of a subset of studies with data on the association of maternal body mass index (BMI, kg/m^2^) with offspring macrosomia or large-for-gestational age (LGA).

**Results:**

A total of 5905 titles/abstracts were screened, 775 full-texts reviewed, and 137 studies synthesized. Maternal anthropometrics were the most frequent risk marker. Meta-analysis demonstrated that women with GDM and overweight/obesity vs. GDM with normal range BMI are at higher risk of offspring macrosomia (13 studies [*n* = 28,763]; odds ratio [OR] 2.65; 95% Confidence Interval [CI] 1.91, 3.68), and LGA (10 studies [*n* = 20,070]; OR 2.23; 95% CI 2.00, 2.49). Lipids and insulin resistance/secretion indices were the most studied non-glycemic biochemical markers, with increased triglycerides and insulin resistance generally associated with greater risk of offspring macrosomia or LGA. Studies evaluating other markers had inconsistent findings as to whether they could be used as precision markers.

**Conclusions:**

Maternal overweight/obesity is associated with greater risk of offspring macrosomia or LGA in women with GDM. Pregnancy insulin resistance or hypertriglyceridemia may be useful in GDM risk stratification. Future studies examining non-glycemic biochemical, genetic, other -omic, or sociocultural precision markers among women with GDM are warranted.

## Plain language summary

Gestational Diabetes (GDM) is high blood sugar that develops during pregnancy and may cause complications. GDM diagnosis is centered on blood sugar levels. Despite everyone receiving standard treatment, the clinical outcomes may vary from one individual to another. This indicates a need to identify factors that may help GDM diagnosis and result in improved classification of those at greatest risk for complications. Here, we systematically analyzed all published evidence for potential markers that could identify those with GDM who have greater risk of complications. We find that high maternal weight is a risk factor for offspring born larger for their gestational age. Other promising markers were identified, but further analysis is needed before they can be applied in the clinic.

## Abstract (web summary)

Francis et al. perform a systematic review and meta-analysis to evaluate studies comparing perinatal outcomes among individuals with gestational diabetes mellitus (GDM). Their review and post hoc analysis find that maternal preconception weight and non-glucose-dependent biochemical markers could be a precision diagnostic approach to reducing variability in clinical outcomes following treatment.

## Introduction

Gestational diabetes (GDM) is the most common metabolic complication of pregnancy with an increasing prevalence consistent with the concomitant global increase in obesity and diabetes^1^. GDM traditionally refers to abnormal glucose tolerance with onset or first recognition during pregnancy, typically diagnosed between 24 and 28 weeks’ gestation^2^. It is associated with maternal and neonatal complications such as hypertensive disorders of pregnancy, offspring large-for-gestational-age (LGA), macrosomia, birth trauma, neonatal respiratory distress, and neonatal hypoglycemia^3^.

Although treating hyperglycemia lowers the risk of maternal and neonatal morbidity, some women with GDM likely would not have had perinatal complications even if left untreated^4,5^, while others still go on to develop complications despite adequate glycemic control^6^. Maternal GDM with obesity (BMI ≥ 30 kg/m^2^) vs. GDM without obesity is associated with a 2- to 4-fold greater risk of macrosomia^7–10^. Recently, differences in perinatal outcomes based on physiologic subtypes of GDM (e.g., insulin-resistant vs. insulin secretion deficient) have been described^11–14^. While the diagnostic criteria for GDM detect dysregulation of glucose metabolism, GDM is increasingly recognized as a heterogeneous condition, which may include sub-phenotypes^6,15^. As such, metabolic variations, beyond glycemic measures among women with GDM may modify its impact on maternal and fetal health^16^.

Several upstream determinants of metabolic health are considered risk factors for GDM and may also be markers by which to stratify risk within the population of women who develop GDM. These risk factors include genetics, higher BMI, prior medical and obstetric history, and socio-demographic factors such as race/ethnicity which may capture differences in societal and environmental factors^17–19^. Prior systematic reviews have focused on the prediction or prevention of GDM^20–23^, have used a normoglycemic group for comparison^24,25^, or have focused on glycemic markers as the sub-phenotyping variable among women with GDM^26^. Despite great interest in risk stratification of women with GDM, efforts to systematically review studies evaluating a spectrum of social, physiological, and biological non-glycemic factors that could identify sub-phenotypes within GDM are lacking.

The Precision Medicine in Diabetes Initiative (PMDI) was established in 2018 by the American Diabetes Association (ADA) in partnership with the European Association for the Study of Diabetes (EASD). The ADA/EASD PMDI includes global thought leaders in precision diabetes medicine who are working to address the burgeoning need for better diabetes prevention and care through precision medicine^27^. As part of the ADA/EASD PMDI effort to comprehensively evaluate the evidence for precision diabetes medicine and inform the 2nd International Consensus Report on Precision Diabetes Medicine^28^, we aimed to review the existing literature to investigate GDM sub-phenotypes and heterogeneity in association with adverse perinatal outcomes. This effort was undertaken to aid in determining whether factors other than traditional glycemic measures could refine the diagnosis of GDM. The following categories of precision markers were included: maternal anthropometrics, clinical or socio-cultural factors, diet and behaviors, non-glycemic biochemical markers, genetics/genomics or other -omics, and fetal biometry.

Our systematic review of 137 studies and 432,825 women with GDM demonstrates that perinatal outcomes vary substantially related to factors that extend beyond glycemia. Prior research has largely focused on the impact of pre-pregnancy overweight or obesity on adverse perinatal outcomes. In a meta-analysis of 10 studies of LGA and 13 studies of macrosomia, we found that the co-occurrence of pre-pregnancy overweight/obesity with GDM was associated with a 2 to 3-fold greater risk of LGA or macrosomia. Furthermore, independent of maternal BMI, those with higher triglycerides or insulin resistance, may be at higher risk of having an offspring born LGA or with macrosomia. Areas that currently require more evidence include investigations of genetics, metabolites, and other novel biomarkers, as well as integration of social, environmental, and behavioral factors. Overall, our systematic review identified critical gaps and future research areas for precision GDM diagnosis and highlighted promising biomarkers that may open the door to non-glycemic treatment targets in GDM.

## Methods

A protocol for this review was registered at PROSPERO (CRD42022316260) on 11 March 2022. *Nota bene*, as part of the diabetes scientific community, ADA/EASD PDMI is committed to using inclusive language, especially in relation to gender. We choose to use gendered terminology throughout the article following the rationale for using female-sexed language in studies of maternal and child health^29^. Additionally, most of the original studies reviewed used “women” as their terminology to describe their population, as GDM per definition is a pregnancy complication, which can only occur in individuals who are assigned female sex at birth. In this review, we use the term “women” throughout, but acknowledge that not all individuals who experienced a pregnancy may self-identify as women.

### Data sources and search strategy

Systematic literature searches were performed in PubMed (https://pubmed.ncbi.nlm.nih.gov/) and EMBASE (https://www.embase.com) databases from their inception to March 2022. These databases were chosen because both can be searched using multiple retrieval approaches such as text word terms in relevant fields and standardized subject terms (controlled vocabulary). Searches were limited to databases that could return results for a combination of these two approaches, a decision informed by the mandatory requirement of the Cochrane Review quality assurance strategy. We searched for observational studies (cohort and case–control) and clinical trials that compared outcomes among women with GDM. The following categories of precision markers were included in the current search: maternal anthropometrics, clinical or socio-cultural factors (i.e., age, race/ethnicity, country of origin), diet and behaviors, non-glycemic biochemical markers (e.g., lipids, insulin, other biomarkers), genetics/genomics or other -omics (e.g., proteomics, lipidomics, metabolomics, metagenomics), and fetal biometry. The search was restricted to studies in adult humans that were published in English. The search strategy and terms are available in Supplementary Note 1. All studies were screened by at least two reviewers and conflicts were resolved by a third independent reviewer. All titles and abstracts were screened for eligibility, and those that were assessed as potentially meeting inclusion/exclusion criteria were selected for full-text evaluation. To help ensure that other important articles were not missed, during full-text evaluation, if a relevant study was referenced, we included those in full-text evaluation as well.

### Inclusion criteria

Studies were considered potentially eligible for inclusion if they met the following criteria: 1) at least 100 participants and a minimum of 30 GDM cases when reporting a continuous precision marker, or 30 cases per GDM subtype, 2) reported outcome data on maternal hypertensive disorders in pregnancy, cesarean delivery, offspring anthropometry at birth (macrosomia, LGA, small-for-gestational-age [SGA]), preterm delivery, birth trauma, metabolic sequelae (e.g., hypoglycemia) or mortality, and 3) presented data in text or tables that allowed for the comparison of outcomes between subtypes of GDM or among women with GDM exclusively for continuous markers. Studies evaluating prevention, treatment, or long-term maternal and offspring prognosis were excluded as they were to be covered by the objectives of complementary systematic reviews led by other PDMI working groups^30–32^.

### Exclusion criteria

As our main goal was to review studies identifying GDM sub-phenotypes beyond glycemia, we excluded studies that only reported on glycemic markers or thresholds (e.g., HbA1c, fasting glucose, oral glucose tolerance test [OGTT] glycemic values), or studies that were focused on assessing differences in outcomes based on timing of glucose measurement.

We also excluded studies that 1) measured the precision marker after GDM diagnosis (e.g., total gestational weight gain [GWG] over the whole pregnancy, or fetal biometry after 32 weeks’ gestation), 2) combined pre-existing diabetes or overt diabetes (based on non-pregnancy glycemic thresholds) with GDM, 3) included women with multi-gestations, or 4) did not contain full-length manuscripts in English.

### Data extraction and quality assessment

Study and sample characteristics were extracted independently by two reviewers and conflicts were resolved by a third reviewer from full-text using a web-based collaboration software platform that streamlines the production of systematic literature reviews (Covidence systematic review software, Veritas Health Innovation, Melbourne, Australia). The following data elements were extracted from each study when available: cohort characteristics (continent, country, study type [hospital/registry/cohort], enrollment years); participant characteristics (age, BMI, the proportion nulliparous); GDM information (sample size, diagnostic criteria or description); timing of precision marker measurement (pre-pregnancy, before or at GDM diagnosis); and perinatal outcomes (maternal, fetal/neonatal).

The risk of bias and overall quality of each study were assessed independently or in duplicate using the Joanna Briggs Institute Critical Appraisal Tool for cohort studies, which was modified specifically for the objectives of the current systematic review^33^ (Supplementary Note 2). We assessed the studies using a ten-question measure and considered studies with two poor quality metrics to be of low quality^34^.

### Data synthesis and meta-analysis

For each category of precision marker, two independent reviewers jointly summarized qualitatively the findings. Given the numerous studies evaluating effect modification by maternal BMI, we performed a post-hoc meta-analysis of studies that reported data that allowed for quantitative measurement of the associations of maternal BMI with offspring LGA or macrosomia among women with GDM.

We pooled odds ratios (ORs) from individual studies to estimate the summary OR with 95% CI for each BMI category using the Dersimonian and Laird random-effects model accounting for both within- and between-study variances^35^. We assessed overall heterogeneity using the Cochran’s Q test and *I*^2^ statistics; *I*^2^ > 75% was considered as evidence of statistical heterogeneity^36^. Subgroup analyses were carried out by study enrollment period (enrollment completed prior to 2010 vs. enrollment from 2010 onwards), quality grade (≥2 poor quality metrics vs. <2 poor quality metrics), and covariate adjustment (yes vs. no). Degree of potential publication bias was evaluated using the Egger’s test and the Begg’s test^37,38^. Meta-analyses were conducted using R (version 4.2.3) and the ‘metafor’ R package (version 4.0-0). A two-sided *p* value of <0.05 was considered statistically significant.

### Reporting summary

Further information on research design is available in the Nature Portfolio Reporting Summary linked to this article.

## Results

### Literature search

**Fig. 1:**
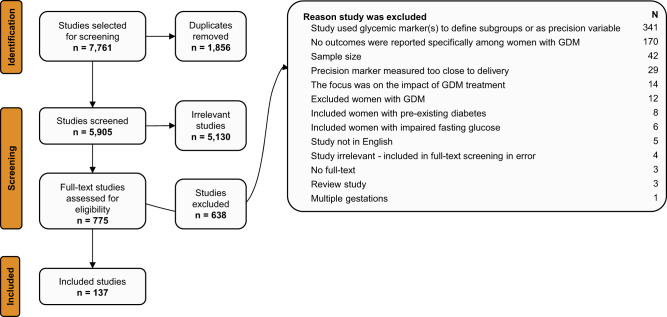
PRISMA systematic review attrition diagram. This shows the flow diagram for the number of references that were identified, screened, and included.

The literature search yielded 5905 non-duplicated abstracts (Fig. 1). After independent review by two investigators for each abstract, 5130 abstracts were excluded. Among the 775 full-text studies reviewed, 638 were excluded based on our study selection criteria, resulting in 137 studies that met the selection criteria. All 137 studies were observational, with no randomized clinical trials. The 137 studies were categorized into three groups depending on the precision markers examined: 1) anthropometry (maternal anthropometry/fetal biometry); 2) biochemical, genetics, -omics markers; and 3) clinical or socio-cultural factors.

### Overall study characteristics

Characteristics of the 137 studies representing a total of 432,156 participants are shown in Supplementary Data 1. The median number of study participants was 587. Of these studies, 68 evaluated maternal anthropometry, 33 evaluated non-glycemic biochemical markers, and 48 evaluated clinical or socio-cultural factors (some studies reported more than one precision marker). Most studies (72%) included pregnancies from 2000 to 2020 and were from geographically diverse regions. The studies were most frequently conducted in China (20%), the US (12%), Australia (7%), and Spain (6%). The most frequent diagnostic criteria for GDM were the 2010 International Association of the Diabetes in Pregnancy Study Groups (IADPSG)^39^/2013 World Health Organization (WHO) criteria^40^.

**Fig. 2:**
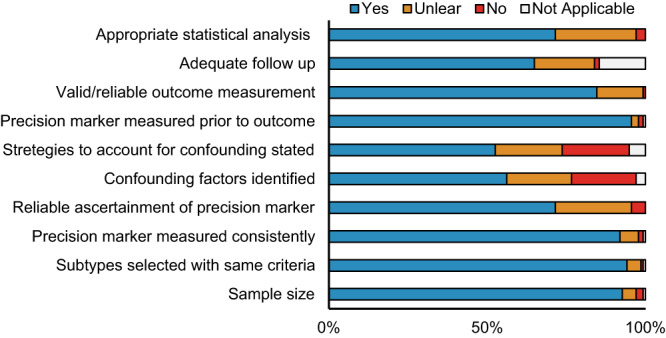
Quality assessment of the included studies by critical appraisal domain. The risk of bias and overall quality of each study was assessed independently or in duplicate using the Joanna Briggs Institute (JBI) Critical Appraisal Tool for cohort studies, which was modified specifically for the objectives of the current systematic review. For each question, a reviewer could indicate “not applicable” (blank filled bars), “yes” (blue filled bars), “unclear” (orange filled bars), “no” (red filled bars). An answer of “yes” indicates less risk of bias and greater quality, and answer of “no” indicates a higher risk of bias and lower quality.

Overall, 45% of the studies had two or more quality assessment domains categorized as low (Fig. 2). The most frequent domain ranked as low was related to confounding; ~40% of studies reported unadjusted effect size estimates. In addition, self-reported data is generally considered to be of low quality, and since many studies ascertained maternal weight and/or BMI using self-reported pre-pregnancy weight, 28% of studies had low-quality rankings on the “ascertainment of precision marker” domain. Other factors that impacted the quality rankings were mostly due to unclear reporting in the manuscripts.

### Studies of anthropometry as a precision marker

Study characteristics—a total of 68 studies of women with GDM described associations of pre-pregnancy overweight and obesity (defined by maternal BMI based on WHO classifications^41^ or region-specific cut-offs) with adverse pregnancy and perinatal outcomes^7–10,42–105^. A small number of studies described the relationship of early gestational weight gain (early GWG) prior to GDM diagnosis (*n* = 4)^49,106–108^, or fetal biometry ultra-sound measures (biparietal, head, abdominal circumference or femur length) before 32 weeks’ gestation (*n* = 9)^42,55,64,87,98,109–112^ with adverse perinatal outcomes. The characteristics of these studies are summarized in Supplementary Data 2. The median number of GDM cases was 594.

Maternal anthropometry—studies evaluating the relationship between maternal BMI and adverse pregnancy outcomes tended to be retrospective hospital record cohort or case-control studies relying on self-reported pre-pregnancy weight. All but nine studies^44,59,65,80,88,94–96,103,113–115^ reported that maternal overweight and obesity were associated with greater risk of at least one adverse perinatal outcome, most commonly offspring LGA or macrosomia.

**Fig. 3:**
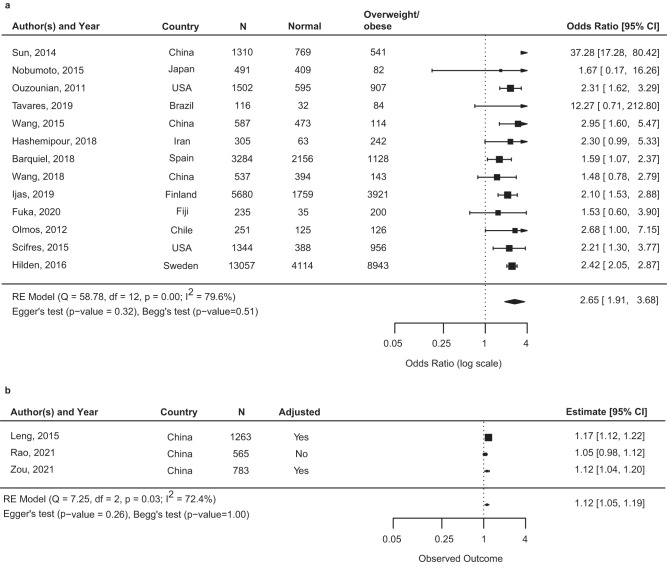
Summary odds ratio (95% CI) of macrosomia for maternal body mass index overweight/obese vs. normal or continuously. Square represents the odds ratio on the log scale; confidence interval (CI). **a** It shows the odds ratio (95% CI) for the association of maternal BMI categorized in overweight/obesity vs. normal range and offspring macrosomia among 13 studies that included a total 28,763 participants. **b** Odds ratio for the association of continuous maternal BMI (per kg/m^2^) and offspring macrosomia among three studies that included 2611 participants. Abbreviations: LGA large-for-gestational age.

**Fig. 4:**
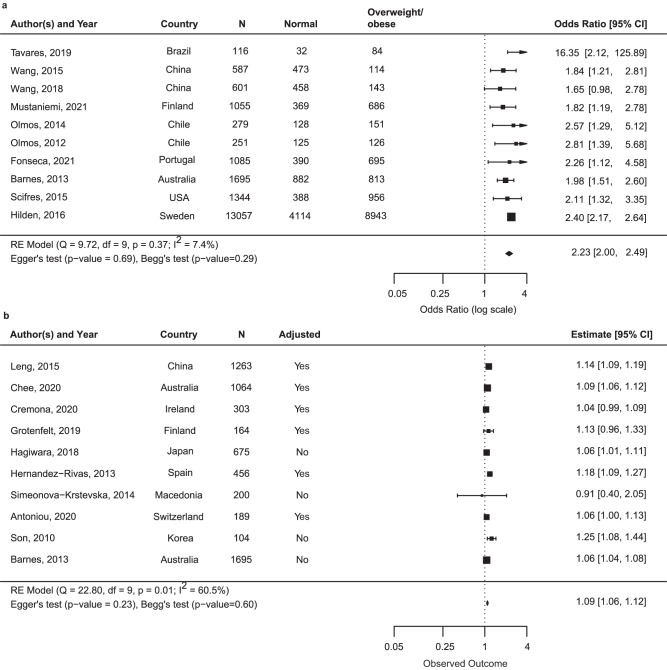
Summary odds ratio (95% CI) of large-for-gestational age for maternal body mass index overweight/obese vs. normal or continuously. Square represents the odds ratio on the log scale; confidence interval (CI). **a** Odds ratio (95% CI) for the association of maternal BMI categorized in overweight/obesity vs. normal range and offspring LGA among 10 studies that included a total 20,070 participants. **b** Odds ratio for the association of continuous maternal BMI (per kg/m^2^) and offspring LGA among 10 studies that included 6113 participants. Abbreviations: LGA: large-for-gestational age.

Thirty-eightstudies reported data that could be meta-analyzed to offer a quantitative assessment of maternal BMI as a precision marker for risk of macrosomia or LGA^7–10,42,45–49,54–56,58,61,66,67,69–73,77,79,81,82,84,88–90,92–94,98,100,101,105,113^. Among women with GDM, the association of maternal BMI and macrosomia was reported in 23 studies (*n* = 34,016)^7–10,45,47,48,56,61,70–73,77,81,84,90,92–94,98,100,113^ and the association of maternal BMI and LGA was reported in 26 studies (*n* = 31,287)^8,10,42,46,49,54,55,58,66,67,69–72,77,79,81,82,84,88–90,93,94,101,105^. Across the 38 studies, there were differences in how the BMI categories were constructed and which category was used as a reference. Pooled estimates from the 13 studies that reported maternal overweight/obesity categories (versus BMI in normal range) and macrosomia (Fig. 3; *n* = 28,763)^7,9,10,45,47,51,61,70,73,81,90,93,94^ and the ten studies that reported maternal overweight/obesity categories (versus BMI in normal range) and LGA (Fig. 4; *n* = 20,070)^10,49,70,79,81,90,93,94,101^ are reported below. Meta-analysis of other categories of BMI and reference groups (e.g., obese vs. non-obese) can be found in Supplementary Fig. 1 and Supplementary Fig. 2.

Macrosomia—the pooled OR from 13 studies of macrosomia (*n* = 28,763)^7,9,10,45,47,51,61,70,73,81,90,93,94^ was 2.65 (95% CI: 1.91–3.68) for overweight/obesity compared to normal BMI with heterogeneity noted across the pooled studies (*I*^2^ > 75%) (Fig. 3). Study-specific ORs based on country of recruitment are presented in each figure. The association between overweight/obesity and macrosomia among women with GDM in subgroup meta-analyses is shown in Supplementary Table 1. Subgroup analysis of studies with low quality (*n* = 3) yielded an OR of 6.44 (95% CI: 0.84-49.42), and among studies of high quality (*n* = 10) the *I*^2^ was 0% indicating study quality as a source of heterogeneity in the full analysis. Subgroup analyses focusing on studies with enrollment during or after 2010 (all of which used IADPSG diagnosis criteria), studies of high quality, or studies with covariate adjustment did not substantially change the results. No indication of publication bias was suggested based on either the Egger’s test (*P* = 0.32) or the Begg’s test (*P* = 0.51). Three studies examined maternal BMI as a continuous variable associated with macrosomia in women with GDM (*n* = 2611)^8,98,113^. A one-unit increment of maternal BMI (kg/m^2^) was significantly associated with an increased risk of macrosomia (OR: 1.12, 95% CI: 1.05–1.19) (Fig. 3).

LGA—the pooled OR from 10 studies of LGA (*n* = 20,070)^10,49,70,79,81,90,93,94,101^ was 2.23 (95% CI: 2.00–2.49) for overweight/obesity vs. normal BMI (Fig. 4). Study-specific ORs based on country of recruitment are presented in each figure. Subgroup analysis, where we stratified studies by enrollment during or after 2010 (all of which used IADPSG diagnostic criteria), or study quality rating, or restricted to studies with covariate adjustment did not produce substantially different results (Supplementary Table 2). No indication of publication bias was suggested based on either the Egger’s test (*P* = 0.69) or the Begg’s test (*P* = 0.29). Ten studies reported associations of continuous BMI with offspring LGA (*n* = 6113)^8,42,49,55,58,66,67,69,88,89^. A one unit increment of maternal BMI (kg/m^2^) was associated with increased odds of LGA (summary OR: 1.09, 95% CI: 1.06–1.12) **(**Fig. 4).

Numerous studies among women with GDM also found associations between maternal overweight/obesity and greater risk of cesarean delivery^44,51,54,64,70,72,73,75,78,92,95,105^ or preeclampsia and other hypertensive disorders of pregnancy^46,47,53,54,70,72,92,99,100,102,104^. Four studies reported an association with neonatal hypoglycemia^56,58,61,62^, two reported an association with a composite outcome of neonatal morbidity and/or admission to NICU^47,96^, and one reported an increased risk of major congenital malformations^63^.

Early gestational weight gain (GWG)—three of four^49,106–108^ studies of early GWG (prior to GDM diagnosis) in women with GDM found positive associations with LGA^49,106,108^, one of which reported that trimester-specific weight gain above the Institute of Medicine Guidelines^116^ was additionally associated with increased risk of preeclampsia and macrosomia^106^.

Fetal biometry—among the nine studies with a fetal biometry ultra-sound measure near the time of GDM diagnosis, six found that larger fetal abdominal^42,55,87,110,112^ or biparietal circumference^98^ was positively associated with greater neonatal size (birthweight, LGA, macrosomia).

### Studies of biochemical, genetics, or -omics as precision markers

Study characteristics—of the studies that reported biochemical, genetic, or -omics (e.g., metabolomic, lipidomic) markers, 14 described associations of lipid classes (triglycerides, total cholesterol, LDL cholesterol, and HDL cholesterol) with adverse pregnancy and perinatal outcomes^7,65,66,74,82,88,89,97–99,113,117–119^. There were 12 studies that described associations of insulin sensitivity/resistance profiles^12,114,115,120–124^ or insulin secretion indices^109,117,125,126^ with perinatal outcomes. A small number of studies subtyped GDM based on adipokines (*n* = 2)^109,127^, metabolomics (*n* = 1)^128^, non-coding RNA (*n* = 2)^129,130^, and variants in candidate genes (*n* = 2)^131,132^. The characteristics of these studies are summarized in Supplementary Data 3, which also includes studies with measurement of other biochemical markers (e.g., proteinuria, platelet count). The median (range) number of GDM cases was 242 (64-2647).

Lipid subclasses—among the 14 studies measuring triglycerides prior to or at the time of GDM diagnosis^7,65,66,74,82,88,89,97–99,113,117–119^, half reported that higher triglycerides positively correlated with increased birthweight or risk of LGA or macrosomia after adjusting for maternal BMI^7,82,88,89,97,98,113^, whereas six reported no association with neonatal size^65,66,74,117–119^, and one reported no association with preeclampsia^99^. Of the studies measuring total, LDL, or HDL cholesterol (*n* = 12)^65,66,74,88,89,97–99,113,115,118,119^, one found a positive association of LDL with LGA^89^, and three reported lower mean HDL levels were associated with LGA^66,74,119^.

Insulin profiles and indices—a variety of methods of calculating insulin resistance/sensitivity and insulin secretory response using timed insulin and glucose during the OGTT for GDM subtyping were described. The homeostatic model assessment of insulin resistance (HOMA-IR or HOMA2-IR) calculated at the time of GDM diagnosis (http://www.dtu.ox.ac.uk/homacalculator/)^133^ was most commonly used. The Matsuda index^134^, modeled using glucose and insulin values across the OGTT, was the most frequent measure of insulin sensitivity. HOMA-B/HOMA-2B (http://www.dtu.ox.ac.uk; homeostatic model assessment of beta-cell function; fasting insulin and fasting glucose model), and the Stumvoll first phase insulin estimate (modeled using timed insulin and glucose values from OGTT)^135,136^ were the most utilized indices defining insulin secretory response. Other indices such as the insulinogenic index and disposition index were utilized rarely^120,122^.

All four studies examining HOMA-IR found that women with GDM and high HOMA-IR (highest quartile or >2.0) had a significantly increased risk of LGA or macrosomia compared to those with GDM and lower HOMA-IR^115,121,124,126^, although in one study the statistical comparison was to normal glucose tolerant women^126^. In two studies, insulin-related measures such as a defect in insulin sensitivity, insulin secretion, or a combination of both were not associated with differences in perinatal outcomes^12,123^. Three studies reported on insulin profiles among participants with and without GDM^114,120,122^. In two of these, participants with GDM who were insulin resistant had higher rates of LGA and macrosomia (where women with GDM without greater than usual insulin resistance had similar rates as women without GDM); however, no statistical tests for significant differences in the outcome rates among the subtypes of GDM were reported^114,122^. A study of insulin secretion peaks during an OGTT found that a delayed insulin secretion peak was associated with increased risk of preeclampsia, LGA, and neonatal hypoglycemia^125^, whereas both a study of insulin levels following a 50 g glucose load and a study of fasting plasma insulin found no association with adverse perinatal outcomes^109,117^.

Adipokines—two studies measured adiponectin, leptin^109,127^, and one additionally measured visfatin^127^. Neither study found that adiponectin or leptin was associated with perinatal outcomes among women with GDM; however, higher visfatin levels were associated with lower risk of LGA^127^.

Metabolomics—a single study utilizing mass spectrometry examined the association of plasma levels of carnitine and 30 acylcarnitines with adverse perinatal complications in women with GDM^128^. Carnitine and acylcarnitine levels together with clinical factors were used to construct a nomogram to predict macrosomia within women with GDM, which resulted in an area under the receiver operating characteristic (AUROC) curve of 0.78.

Non-coding RNAs—two studies examined the association of different classes of non-coding RNAs with various adverse pregnancy outcomes^129,130^. One study of circulating long non-coding RNAs (lncRNAs) measured in 63 women with GDM found that including XLOC_014172 and RP11-230G5.2 in a prediction model for macrosomia resulted in an AUROC curve of 0.962^129^. In a study of high or low plasma levels of circular RNA circATR2, high circATR2 was associated with higher rates of prematurity, miscarriage, intrauterine death, fetal malformations, intrauterine infection and hypertension but not macrosomia or fetal distress^130^.

Genetic studies—we did not identify any genome-wide-association studies reporting on GDM subtypes. Two studies used a candidate gene approach to subtype women with GDM based on their genotype and examine associations with pregnancy outcomes^131,132^. One study of a variant in the patatin-like phospholipase-3 (*PNPLA3*)/adiponutrin gene (rs738409 C.G) found that carrying the G allele (*n* = 96) compared with being a CC homozygote (*n* = 104) was associated with lower fasting insulin, insulin resistance, and LGA^132^. In a study of SNP 45TG in exon 2 of the adiponectin gene, the G allele and GG + TG genotypes were associated with GDM, lower adiponectin levels, and among the women with GDM, greater incidence of macrosomia and neonatal hypoglycemia compared to the TT group^131^.

### Studies of clinical and sociocultural factors as precision markers

Study characteristics—of the studies reporting associations of clinical, sociocultural factors or composites of multiple risk factors among women with GDM^55,59,60,65,69,78,80,85,86,96,97,103,104,113,137–171^, 14 compared differences in adverse perinatal outcomes between different races, ethnicities, or countries of origin^69,96,147–150,152,154–160^, and six included multiple risk factors as a composite variable^78,86,141,144,162,163^. Four studies investigated psychological factors, and five reported on concomitant presence of pre-eclampsia or hypertensive disorders of pregnancy in women with GDM. The characteristics of these studies are summarized in Supplementary Data 4. Half of the studies included pregnancies from 1990-2009, with four studies from the 1980s. A third of studies diagnosed GDM using 2010 IADPSG criteria, and 20% did not report diagnostic criteria. The median (range) number of GDM cases in these 48 studies was 950 (100–170,572).

Race/ethnicity—studies have reported various findings comparing outcomes in women with GDM from different races or ethnicities^69,96,147–150,152,154–160^. In the US, women with GDM who identified as Black or African American were at higher risk of perinatal complications, including fetal death^148,154^. Findings were inconsistent regarding risk of reported complications in women with GDM who identified as Hispanic (versus non-Hispanic): two studies did not find major differences in adverse outcomes^149,158^, while one large study reported a higher rate of preterm birth^148^. In Hawaii, White women with GDM were at greater risk of macrosomia compared to other race/ethnicity groups (Hawaiian/Pacific Islander, Filipina, or other Asian women)^152^. Several studies in Australia, US, and Canada comparing women with GDM from different race/ethnicity groups found that women who identified as Asian were less likely to have LGA offspring (compared to White-identified women)^147,150,154,155,159,161^. In two Canadian studies, women with GDM from First Nations or Indigenous groups were at higher risk of perinatal complications^150,157^.

Clinical, and co-existing medical factors and conditions—many studies that met our inclusion criteria also reported the association of other factors such as prior history of GDM, macrosomia, polycystic ovary syndrome (PCOS), or family history of diabetes with risk of perinatal adverse outcomes among women with GDM. Given that these risk factors were not pre-specified in our search, our summary of these studies should be considered as a scoping (rather than systematic) review.

Of the studies examining multiple clinical or sociocultural factors (e.g., BMI, maternal age, prior GDM pregnancy), four found that the presence of one or more risk factors was associated with greater neonatal size (birthweight percentile, LGA, macrosomia), compared to women with GDM and no risk factors^78,86,141,162^. Two of these studies reported a higher risk of cesarean delivery^78,86^. One study reported that GDM with one or more risk factors was associated with cesarean delivery and not neonatal size^144^, and another found no difference in perinatal outcomes among women with or without risk factors^163^.

In general, there was no consistent association of maternal age, parity, prior GDM, or family history of diabetes with risk of adverse perinatal outcomes in women with GDM. Studies that identified co-existing medical conditions (e.g., PCOS, preeclampsia, hypertensive disorders of pregnancy, infertility treatment) showed that PCOS was a marker for higher risk of preeclampsia^140,145^. Co-existing preeclampsia or hypertensive disorder of pregnancy in women with GDM was associated with smaller size at birth compared to GDM pregnancies without preeclampsia or hypertensive disorder of pregnancy^57,80^.

Four studies reporting on psychological factors found that worse depression, anxiety, or diabetes distress scores^146,151,153,164^ were markers of greater risk of various adverse perinatal outcomes, or were related to differences in neonatal size^146,151,153^. There was a lack of studies evaluating diet or physical activity as risk markers among women with GDM. Three studies included other risk factors (e.g., fetal sex, seasonality of conception, epicardial fat)^167,170,171^ with findings that will need replication before conclusions can be drawn.

## Discussion

Our systematic review of 137 studies and 432,825 women with GDM demonstrates that perinatal outcomes vary substantially related to factors that extend beyond glycemia. Prior research has largely focused on the impact of pre-pregnancy overweight or obesity on adverse perinatal outcomes. In a meta-analysis of 10 studies of LGA (*n* = 20,070) and 13 studies of macrosomia (*n* = 28,763), we found that the co-occurrence of pre-pregnancy overweight/obesity with GDM was associated with a 2 to 3-fold greater risk of LGA or macrosomia. Notably, even across the spectrum of maternal size, a one-unit increase in BMI is associated with a 9% greater risk of LGA and a 12% greater risk of macrosomia. Furthermore, independent of maternal BMI, those with higher triglycerides or insulin resistance, may be at higher risk of having an offspring born LGA or with macrosomia. Studies reporting on genetics and ‘omics were scarce, and we could not draw conclusions on these potential precision markers. There was inconsistent evidence that individual maternal clinical and sociocultural factors were associated with greater risk of perinatal complications.

### Anthropometry as a precision marker

Findings from our meta-analysis provide strong evidence that women with GDM and overweight/obesity compared to women with GDM and BMI in the normal range are at greater risk of fetal overgrowth. Although assessment of the relative contribution of maternal glycemia versus obesity to adverse pregnancy and perinatal outcomes was beyond the scope of this review, the risks associated with obesity and GDM are likely to be additive^54^. Current evidence suggests metabolic alterations that accompany obesity increase the risk of adverse perinatal outcomes^172^. This underscores the need to better refine the phenotyping of women with GDM based on lipids, insulin resistance, and other metabolic alterations that may contribute to fetal overgrowth.

Fetal biometry is not a novel precision marker of overgrowth risk and arguably reflects the consequences of GDM. Nevertheless, few research studies have evaluated a combination of early ultrasound fetal growth biometry with other metabolic data, in association with, or prediction of, adverse perinatal outcomes. These studies may help identify early metabolic biomarker profiles (and, therefore, targets) of birth size.

### Biochemical, genetics, or -omics as precision markers

Most studies examining lipids in association with adverse perinatal outcomes have measured a standard lipid panel that includes three measures of cholesterol levels (total, LDL and HDL cholesterol) and triglycerides. Half of the studies reported higher triglycerides, independent of BMI, were associated with macrosomia or LGA, with fewer studies finding that higher LDL or lower HDL was associated with neonatal size. Data on the association of triglycerides with neonatal size in women with GDM align with a recent meta-analysis of studies in the general pregnant population that higher triglycerides were associated with increased birthweight and higher risk of LGA and macrosomia^173^. However, not all studies included in our review reported positive associations, and many factors, such as differences in timing of blood collection and variability in the distribution of characteristics across studies, could explain inconsistencies. Few studies examined the joint effects of multiple lipid subclasses, and future studies should include other and more detailed lipid measures to further clarify the mechanisms leading to fetal overgrowth (which lipids or lipid fractions, placental transfer, etc.) so novel therapeutic approaches can be developed and tested.

Many studies reported data on insulin profiles among women with GDM but made statistical comparisons to normal glucose-tolerant women. In general, it appears that women with GDM who have greater insulin resistance are at increased risk of fetal overgrowth and LGA. Subtyping by the presence or absence of insulin resistance and deficiency was described by Powe et al. in 2016, but this paper was not included in our review because of small GDM sub-group sample sizes^11^. Many of the subsequent studies included here used a similar insulin resistance/insulin deficiency classification rubric and referred to Powe et al. as part of the rationale or methodology. There are inadequate data to determine whether a predominant defect in insulin secretion without excess insulin resistance is related to adverse perinatal outcomes. Insulin sensitivity or resistance in pregnancy can be estimated using insulin or C-peptide and glucose values at multiple time-points during the standard OGTT (e.g., using the Matsuda formula which has been validated in pregnancy); however, the studies that calculated insulin resistance using HOMA-IR, which simply requires fasting insulin and glucose values, found that this index was a marker for higher rates of LGA or macrosomia in women with GDM, making it an appealing simple biochemical marker^115,121,124,126^. If GDM subtyping based on insulin physiology is to be translated clinically, there is a need for laboratory standardization of insulin (or C-peptide) assays to support establishing clinical thresholds.

Given the role of adiponectin as an insulin sensitizer^174^ and leptin as modulator of food intake and energy expenditure^175^, as well as the robust body of data tying maternal adiposity to pregnancy outcomes in GDM, it is surprising that our review only identified two studies that reported associations between adipokines and adverse perinatal outcomes among women with GDM. It is difficult to assess if this reflects a publication bias where null findings have been excluded or a true lack of research in this area. Future studies assessing adipose-derived peptides as precision markers among women with GDM should also consider additional effect modification by insulin sensitivity or maternal adiposity. This latter point may be particularly relevant as previous studies of adipokines in general pregnancy have reported effect modification by maternal BMI^176,177^.

No studies that met our inclusion criteria included measures of branched-chain amino acids, which have been implicated in diabetes risk and complications both within and outside of pregnancy^178,179^. Although we recognize that pregnancy cohorts not restricted to GDM have found associations of amino acids with glucose metabolism and perinatal outcomes^180–184^; whether amino acid subclasses or indeed hormonal profiles might be used as potential precision markers among women with GDM that identify increased risk of adverse perinatal outcomes has not been adequately studied and future research in this area is needed.

Studies performed to date attempting to identify genetic markers that predict adverse outcomes among women with GDM are not only limited in number but have major methodologic limitations. First, the identified studies have had small sample sizes and were performed in homogenous populations, without replication in independent cohorts. Moreover, reviewed genetic studies used a targeted approach examining either a single or limited number of variants/molecules. Future studies among larger diverse populations are needed for genome-wide association studies. None of the studies included in our review focused on metagenomics.

### Clinical and sociocultural factors as precision markers

Certain racial/ethnic groups (such as Asian, First Nations/Indigenous, Hispanic) have been observed to have an increased risk of GDM. In the current review, being part of a minoritized race/ethnicity group was associated with adverse outcomes only in some instances. In studies where differences were observed, they mirrored patterns of health disparities reflecting different perinatal complication rates in the general population^185^. We note that these racial and ethnic categories and their relationship with outcomes are highly dependent on the overall social context (countries or regions) and may reflect experiences of racism, some aspects of culture, socioeconomic status, and many other factors that influence health outcomes such as diet and environmental pollutants or toxicants^17–19^. Studies that met our inclusion criteria did not directly investigate some of the correlates of race and ethnicity (e.g., diet, environmental exposures). We encourage future studies to carefully consider and collect data on the sociocultural influences and other correlates of race and ethnicity, and separately investigate genetic similarity, ancestry, or the inequities related to racism.

### Limitations

It is important to highlight that in our review all the studies that met the inclusion/exclusion criteria were observational in design, and thus inadequate to provide the level of evidence required to change clinical practice currently. Different criteria for diagnosing GDM have been adopted at different time periods and across different geographic regions, which ultimately has led to women with differing degrees of hyperglycemia and maternal/fetal risks being diagnosed with GDM^186,187^. In our summary of the literature, IADPSG was the most common reported diagnostic criteria in the studies that were included in our systematic review. We included all studies meeting pre-specified inclusion/exclusion criteria regardless of differences in diagnostic approach. We evaluated non-glycemic precision markers that could reliably distinguish at-risk sub-phenotypes irrespective of the timing, threshold value, or number of above-threshold values. In the current review, the meta-analysis estimates of maternal BMI as a precision marker of macrosomia or LGA risk were similar regardless of diagnostic criteria or time period of study enrollment. We note that not all articles provided details on the definition of macrosomia or LGA, and therefore, this may have introduced some error in the meta-analysis estimates.

In our review, there was an inclusion criterion that manuscripts be published in English, which somewhat limits the global scope of included studies and representation of some regions of the world (e.g., no studies from India were included). Given the increasing prevalence of GDM and risk of diabetes, studies of the utility of precision markers for diagnosing GDM from different regions are needed. We excluded studies with less than 100 participants (and <30 GDM cases), as it would be difficult to draw sound conclusions from studies with fewer participants. However, this undoubtedly limited the inclusion of pilot studies or with smaller sample size, which could have limited the diversity of populations included. Lastly, the GDM diagnosis PMDI working group is a partnership that was initiated by the ADA and EASD which invited GDM experts from across the globe. Although we are an international working group of clinical and research scientists, our expertise may not completely capture the vast global spectrum of scientific and clinical work related to GDM. We hope this initiative will spark additional collaborations and multinational efforts that will continue to contribute to precision initiatives to help identify successes and opportunities in tailored GDM diagnostics.

### Future directions

Our systematic review has identified several major areas for further research. There remains a need for mechanistic studies to provide an understanding of why the identified precision biomarkers are associated with an increased risk of adverse pregnancy outcomes. Replication studies of any potential biomarkers are needed in large and diverse populations across the world, and these should be accompanied by work to standardize the laboratory analysis of biomarkers used to diagnose subtypes of GDM at certain thresholds. Additionally, it will be worthwhile to leverage the information provided herein to investigate whether the identified precision markers for identifying at-risk GDM subtypes are dependent on the criteria used for GDM diagnosis. Multinational studies measuring environmental and behavioral factors such as dietary intake, physical activity, differences in socioeconomic opportunity, and neighborhood characteristics are needed, given their potential impact on perinatal outcomes among women with GDM. Moreover, large studies including participants from many regions across the world with measurements of genetic variants and multi-omics that integrate clinical and sociocultural data are needed and could provide insight into the determinants and causal pathways of heterogeneity within GDM and its outcomes. This may require applying approaches often used in systems biology, machine learning, or in the aggregation and analysis of large datasets from different sources using methods such as multilayer networks and clustering^188^.

For future clinical implications, some of the next questions are: if precision markers such as insulin resistance or higher triglycerides are part of causal pathways that lead to adverse outcomes, can we directly target them safely in pregnancy? Although somewhat controversial, Metformin targets insulin resistance and is accepted in some contexts for use during pregnancy; however, well-designed clinical trials for insulin-resistant GDM women are needed to accurately estimate the benefit-to-risk ratio. There is a need to test whether dietary approaches, supplements, or other therapeutics can effectively and safely reduce triglycerides among women with GDM. Thus, scientific identification of the physiological and mechanist attributes that lead to pregnancy-induced excessive insulin resistance and triglycerides, with linkage to prognostic differences, will improve the development of novel therapeutic options that are targeted to the causal pathways in pregnancy.

## Conclusions

We conducted a systematic review to identify potential precision markers that would refine the diagnosis of GDM and identify women with GDM at higher risk of perinatal complications. The results of our meta-analyses that included over 20,000 women demonstrated that a higher maternal BMI in women with GDM is a marker for risk of offspring LGA or macrosomia. Other promising precision markers include maternal triglycerides and insulin resistance indices (e.g., HOMA-IR); however, these biomarkers require additional replication and development of standardized clinical laboratory assays before implementation in clinical practice. Areas that currently require substantially more evidence include investigations of genetics, metabolites, and other novel biomarkers, as well as integration of social, environmental, and behavioral factors. Advances in computing and the promotion of cross-disciplinary team science may be one approach for addressing these gaps and future directions. Overall, our systematic review identified critical gaps and future research areas for precision GDM diagnosis and highlighted promising biomarkers that may open the door to non-glycemic treatment targets in GDM.

### Supplementary information

Description of Additional Supplementary Files

Supplementary Information

Supplementary Data 1

Supplementary Data 2

Supplementary Data 3

Supplementary Data 4

Supplementary Data 5

Reporting Summary

## Data Availability

Complete lists of the publications where data were extracted for this study are provided in Supplementary Data 1. Source data for Fig. 2 are available in Supplementary Data 5. Additional information is available via contact with the corresponding author. Data for meta-analyses are available via contact with Dr. Jiaxi Yang.
