# Supplementary material for: Refining the diagnosis of gestational diabetes mellitus: a systematic review and meta-analysis
Source: Commun Med (Lond). 2023 Dec 18;3:185. doi: 10.1038/s43856-023-00393-8 (PMC10728189; doi:10.1038/s43856-023-00393-8)
Supplement: Supplementary file 1 — Description of Additional Supplementary Files [file 43856_2023_393_MOESM1_ESM.pdf]

Description of Additional Supplementary Files

File Name: Supplementary Data 1
Description: The characteristics of the studies included in the systematic review. Footnotes:
1 Multinational: US, Canada, West Indies, UK, Israel, Singapore, Thailand, China, Australia
2 Multinational: Spain, UK, Austria, Belgium, Denmark, Italy, Ireland, Poland, Netherlands
3 Multinational: Bellflower, CA, US; Cleveland, OH, US; Brisbane, QLD, Australia; Newcastle, NSW, Australia; Hong Kong, China
4 Multinational: New Zealand & Australia
5 Multinational: HAPO (Cleveland, Bellflower, Brisbane, Newcastle, Hong Kong)
NR: not reported

File Name: Supplementary Data 2
Description: Characteristics of studies that included anthropometry
Footnotes:
a BMI is reported as mean/median depending on how the study originally reported it or else as %; ¥ % of women with GDM with a BMI ≥ 30.0 kg/m2 ; § % of women with GDM with a BMI ≥ 25.0 kg/m2
Multinational a: USA, Canada, West Indies, UK, Israel, Singapore, Thailand, China, Australia

File Name: Supplementary Data 3
Description: Characteristics of studies included as biochemical, genetics, -omics
Footnotes: a BMI is reported as mean/median depending on how the study originally reported it or else as % : ¥ % of women with GDM with a BMI ≥ 30.0 kg/m2 ; § % of women with GDM with a BMI ≥ 25.0 kg/m2
Multinational a: Bellflower, CA, USA; Cleveland, OH, USA; Brisbane, QLD, Australia; Newcastle, NSW, Australia; Hong Kong, China
Multinational b: HAPO (Cleveland, Bellflower, Brisbane, Newcastle, Hong Kong)
Multinational c: Spain, UK, Austria, Belgium, Denmark, Italy, Ireland, Poland, Netherlands

File Name: Supplementary Data 4
Description: Characteristics of studies included as clinical or sociocultural risk factors
Footnotes: a BMI is reported as mean/median depending on how the study originally reported it or else as %; ¥ % of women with GDM with a BMI ≥ 30.0 kg/m2; § % of women with a GDM with a BMI ≥ 25.0 kg/m2

File Name: Supplementary Data 5
Description: Dataset used to generate Figure 2. (quality assessment of the included studies by critical appraisal domain). The risk of bias and overall quality of each study was assessed independently or in duplicate using the Joanna Briggs Institute (JBI) Critical Appraisal Tool for cohort studies, which was modified specifically for the objectives of the current systematic review. For each question, a reviewer could indicate “not applicable” , “yes”, “unclear”, “no”. An answer of “yes” indicates less risk of bias and greater quality, and answer of “no” indicates a higher risk of bias and lower quality.
