# Supplementary material for: Refining the diagnosis of gestational diabetes mellitus: a systematic review and meta-analysis
Source: Commun Med (Lond). 2023 Dec 18;3:185. doi: 10.1038/s43856-023-00393-8 (PMC10728189; doi:10.1038/s43856-023-00393-8)
Supplement: Supplementary file 2 — Supplementary Information [file 43856_2023_393_MOESM2_ESM.pdf]

Title: Refining the diagnosis of gestational diabetes mellitus: a systematic review and meta-analysis

Authors: Ellen C. Francis [1], Camille E. Powe [2], William L. Lowe, Jr. [3], Sara L. White [4], Denise M. Scholtens [5], Jiaxi Yang [6,7,8], Yeyi Zhu [9], Cuilin Zhang [6,7,8,10], Marie-France Hivert [2,11], Soo Heon Kwak [12], Arianne Sweeting [13], ADA/EASD PMDI Consortium

Contents

Supplementary Note 1: Search strategy 2

Supplementary Note 2: Modified Joanna Briggs Institute critical appraisal checklist for cohort studies........ 11

Supplementary Figure 1: Summary odds ratio (95% CI) of macrosomia for maternal body mass index categories........ 14

Supplementary Figure 2: Summary odds ratio (95% CI) of large-for-gestational age (LGA) for maternal body mass index categories 15

Supplementary Table 1: Subgroup meta-analyses of overweight/obese vs. normal BMI, and continuous BMI: per unit increase and association with macrosomia........ 16

Supplementary Table 2: Subgroup meta-analyses of overweight/obese vs. normal BMI, and continuous BMI: per unit increase and association with large-for gestational age........ 17

Supplementary Note 1: Search strategy

GDM Precision diagnosis

Search strategy Embase (Elsevier)

Date of search: 211005, updated 211202, 220210, 220221, 220223, 220302, 220321

Research question 1

#1 'pregnancy diabetes mellitus'/exp

43097 records

#2 'gestational diabetes':ab,ti OR gdm:ab,ti OR 'pregnancy induced diabetes':ab,ti OR 'pregnancy-induced diabetes':ab,ti

29110 records

#3 #1 OR #2

46421 records

1A How can the diagnosis of GDM be refined?

BMI, biomarkers, phenotypes, age, ethnic*, sociocultural, behavioral factors, diet, exercise/physical activity block

#4 'phenotype'/exp OR 'genetic heterogeneity'/exp OR 'timing'/exp OR 'severity'/exp OR 'insulin resistance'/exp OR 'insulin sensitivity'/exp OR 'insulin release'/exp

1267188 records

#5 phenotyp*:ab,ti OR heterogeneity:ab,ti OR timing:ab,ti OR severity:ab,ti OR 'clinical characteristics':ab,ti OR 'insulin resistance':ab,ti OR 'insulin sensitivity':ab,ti OR 'secretory defect':ab,ti

2239071 records

#6 #4 OR #5

2813362 records

Biomarkers

#7 'biological marker'/exp OR 'biological marker*':ab,ti OR biomarker*:ab,ti

610910 records

Lipids

#8 'triacylglycerol'/exp OR 'very low density lipoprotein'/exp OR 'cholesterol'/exp OR 'lipoprotein'/exp OR 'high density lipoprotein'/exp OR 'apolipoprotein'/exp OR 'hyperlipidemia'/exp OR 'dyslipidemia'/exp OR 'fatty acid'/exp OR 'glycolysated protein' OR 'lipid'/exp OR lipid*:ab,ti OR triacylglycerol:ab,ti OR triglyceride:ab,ti OR vldl:ab,ti OR 'very low density lipoprotein':ab,ti OR cholesterol:ab,ti OR lipoprotein*:ab,ti OR hdl:ab,ti OR 'high density lipoprotein':ab,ti OR hyperlipidemia:ab,ti OR apolipoprotein*:ab,ti OR 'non hdl':ab,ti OR lipidemia*:ab,ti OR lipemia*:ab,ti OR lipemic:ab,ti OR dyslipidemia:ab,ti OR 'fatty acid*':ab,ti OR 'glycolysated protein*':ab,ti

2247395 records

Amino acids

#9 'branched chain amino acid'/exp OR 'creatine'/exp OR 'leucine'/exp OR 'isoleucine'/exp OR 'valine'/exp OR 'alanine'/exp OR 'glutamine'/exp OR 'glutamic acid'/exp OR 'betaine'/exp OR 'aspartic acid'/exp OR 'tyrosine'/exp OR 'glutathione'/exp OR 'serine'/exp OR 'threonine'/exp OR 'histidine'/exp OR 'tryptophan'/exp OR 'glycine'/exp OR 'amino acid'/exp OR 'proteomics'/exp OR proteomic*:ab,ti OR 'branched chain amino acids':ab,ti OR bcaa:ab,ti OR creatine:ab,ti OR leucine:ab,ti OR isoleucine:ab,ti OR valine:ab,ti OR alanine:ab,ti OR glutamine:ab,ti OR glutamate:ab,ti OR betaine:ab,ti OR aspartate:ab,ti OR tyrosine:ab,ti OR glutathione:ab,ti OR serine:ab,ti OR threonine:ab,ti OR histidine:ab,ti OR tryptophan:ab,ti OR glycine:ab,ti OR 'amino acids':ab,ti

2748236 records

Inflammation

#10 'cytokine'/exp OR 'c reactive protein'/exp OR 'high sensitivity c reactive protein'/exp OR 'tumor necrosis factor'/exp OR 'mcp1 protein'/exp OR 'chemokine'/exp OR 'inflammation'/exp OR inflammation:ab,ti OR cytokine*:ab,ti OR interleukin*:ab,ti OR il*:ab,ti OR crp:ab,ti OR 'high-sensitivity crp':ab,ti OR 'high sensitivity crp':ab,ti OR 'c-reactive protein':ab,ti OR 'hs crp':ab,ti OR 'tnf alpha':ab,ti OR 'tumor necrosis factor alpha':ab,ti OR 'mcp 1':ab,ti OR 'monocyte chemoattractant protein 1':ab,ti OR chemokine:ab,ti

6650634 records.

Adipokines or adipocytokines

#11 'adiponectin'/exp OR 'leptin'/exp OR 'retinol binding protein 4'/exp OR 'adipocytokine'/exp OR adiponectin:ab,ti OR leptin:ab,ti OR rbp4:ab,ti OR 'retinol binding protein 4':ab,ti OR adipokine*:ab,ti OR adipocytokine*:ab,ti

101544 records.

Hormones

#12 'sex hormone binding globulin'/exp OR 'estrogen'/exp OR 'hydrocortisone'/exp OR 'prolactin'/exp OR 'progesterone'/exp OR 'somatomedin'/exp OR 'hormone'/exp OR 'hormone* sex hormone-binding globulin':ab,ti OR shbg:ab,ti OR estrogen:ab,ti OR oestrogen:ab,ti OR cortisol:ab,ti OR prolactin:ab,ti OR progesterone:ab,ti OR 'insulin-like growth factor':ab,ti OR igf:ab,ti

799750 records.

Placenta-derived

#13 'human placenta lactogen'/exp OR 'placental growth hormone'/exp OR 'placenta derived':ab,ti OR 'human placental lactogen':ab,ti OR hpl:ab,ti OR 'human chorionic somatomammotropin':ab,ti OR hsc:ab,ti OR 'pregnancy-associate plasma protein a':ab,ti OR 'papp a':ab,ti OR 'placental growth hormone':ab,ti OR pgh:ab,ti

34650 records

Thyroid

#14 'thyroid gland'/exp OR 'thyroglobulin antibody'/exp OR 'thyroid peroxidase antibody'/exp OR 'free thyroxine index'/exp OR 'free liothyronine index'/exp OR 'thyrotropin'/exp OR thyroid*:ab,ti OR 'thyroid stimulating hormone':ab,ti OR thyrotropin:ab,ti OR tsh:ab,ti OR 'thyroglobulin antibody':ab,ti OR 'thyroid peroxidase antibody':ab,ti OR 'free t4':ab,ti OR 'free t3':ab,ti

319602 records.

Metabolomics

#15 'butyric acid'/exp OR 'acetylcarnitine'/exp OR 'acylcarnitine'/exp OR 'carnitine'/exp OR 'lactic acid'/exp OR 'metabolomics'/exp OR metabolomics:ab,ti OR butyrate:ab,ti OR 'butyric acid':ab,ti OR acetylcarnitine:ab,ti OR acylcarnitine:ab,ti OR carnitine:ab,ti OR lactate:ab,ti OR 'lactic acid':ab,ti

309723 records

Genetics

#16 'genome-wide association study'/exp OR 'single nucleotide polymorphism'/exp OR 'genetic variation'/exp OR 'genetic risk score'/exp OR 'microrna'/exp OR 'genetics'/exp OR 'genomics'/exp OR genetics*:ab,ti OR genom*:ab,ti OR 'genome-wide association study':ab,ti OR gwas:ab,ti OR 'single nucleotide variation':ab,ti OR 'single nucleotide polymorphism':ab,ti OR snp:ab,ti OR 'genetic variation':ab,ti OR 'genetic risk score':ab,ti OR 'genotype risk score':ab,ti OR grs:ab,ti OR 'polygenic risk score':ab,ti OR 'polygenic score':ab,ti OR prs:ab,ti OR 'micro rna':ab,ti OR mirna:ab,ti

2236966 records

Epigenetics

#17 'cpg island'/exp OR 'dna methylation'/exp OR 'methylome'/exp OR 'epigenetics'/exp OR epigenetics:ab,ti OR 'cpg ilands':ab,ti OR 'dna methylation':ab,ti OR epigenomes:ab,ti OR methylome:ab,ti

170397 records

Exosomes

#18 'exosome'/exp OR exosome*:ab,ti
43335 records

All biomarkers combination
#19 #7 OR #8 OR #9 OR #10 OR #11 OR #12 OR #13 OR #14 OR #15 OR #16 OR #17 OR #18

12968907 records

#20 age:ab,ti OR ethnic*:ab,ti OR white:ab,ti OR caucasian:ab,ti OR asian:ab,ti OR african:ab,ti OR social:ab,ti OR economic:ab,ti OR socioeconomic:ab,ti OR socio-economic:ab,ti

5527104 records

#21 'exercise'/exp OR 'physical activity'/exp

795039 records

#22 exercise:ab,ti OR physical activity:ab,ti

340721 records

#23 #21 OR #22

954221 records

#24 ‘body mass’/exp OR 'body composition'/exp OR 'anthropometry'/exp OR 'obesity'/exp

1058932 records

#25 ‘BMI’:ab,ti OR ‘bodymass’:ab,ti OR ‘body mass’:ab,ti OR ‘body mass index’:ab,ti OR 'body composition':ab,ti OR anthropometry:ab,ti OR obesity:ab,ti OR overweight:ab,ti OR obes*:ab,ti OR 'excess body weight':ab,ti OR 'excess body fat':ab,ti OR 'excess weight':ab,ti OR 'body weight':ab,ti OR lean:ab,ti OR fat:ab,ti OR 'muscle mass':ab,ti

1405134 records

#26 #24 OR #25

1696955 records

#27 'diet therapy'/exp OR 'diet'/exp

704652 records

#28 (diet:ab,ti OR food:ab,ti OR nutriti*:ab,ti OR 'diet therapy':ab,ti OR 'diet intervention':ab,ti OR eating habit*:ab,ti) AND (composition:ab,ti OR pattern*:ab,ti OR matrix*:ab,ti OR 'percent calories':ab,ti)

11381 records

#29 #27 OR #28

711586 records

#30 'health behavior'/exp

459660 records

#31 'behavio* health':ab,ti OR behavi*:ab,ti

1623947 records

#32 #30 OR #31

1967208 records

Combination phenotype/biomarkers/age-ethnicity-social/lifestyle factors

#33 #6 OR #19 OR #20 #23 OR #26 OR #29 OR #32

19415016 records

Subclassification block

#34 'subtype*':ab,ti OR 'subclassif*':ab,ti OR 'stratif*':ab,ti OR 'subgroup*':ab,ti

1033219 records

Risk block/prediction block RQ1

#35 prognos*:ab,ti OR progress*:ab,ti OR predict*:ab,ti OR model*:ab,ti OR nomogram:ab,ti OR statistical:ab,ti OR score*:ab,ti OR risk*:ab,ti OR 'risk score':ab,ti OR 'risk categor*':ab,ti OR 'risk factor*':ab,ti OR 'risk assessment':ab,ti OR algorithm*:ab,ti OR equation*:ab,ti OR precision:ab,ti OR personali*:ab,ti

11779846 records.

#36 #34 OR #35

12001590 records

Perinatal outcomes block

#37 'birth weight'/exp OR 'large for gestational age'/exp OR 'small for date infant'/exp OR 'cesarean section'/exp OR 'neonatal respiratory distress syndrome'/exp OR 'neonatal respiratory failure'/exp OR 'hypoglycemia'/exp OR

'polycythemia'/exp OR 'macrosomia'/exp OR 'congenital malformation'/exp OR 'birth injury'/exp OR 'hyperbilirubinemia'/exp OR 'perinatal mortality'/exp OR 'perinatal morbidity'/exp OR 'newborn mortality'/exp OR 'newborn morbidity'/exp OR 'fetus death'/exp

1429308 records

#38 (perinatal:ab,ti OR pregnancy:ab,ti OR neonatal:ab,ti OR fetal:ab,ti OR foetal:ab,ti) AND outcome*:ab,ti OR birthweight:ab,ti OR 'birth weight':ab,ti OR 'large for gestational age':ab,ti OR lga:ab,ti OR 'small for gestational age':ab,ti OR 'small-for-gestational-age':ab,ti OR sga:ab,ti OR preterm:ab,ti OR 'cesarean delivery':ab,ti OR cs:ab,ti OR 'neonatal respiratory morbidity':ab,ti OR 'neonatal respiratory stress':ab,ti OR hypoglycemi*:ab,ti OR polycythemi*:ab,ti OR macrosomi*:ab,ti OR 'congenital anomal*':ab,ti OR 'congenital malformation* birth injur*':ab,ti OR hyperbilirubinemia:ab,ti OR ((intrauterine:ab,ti OR fetal:ab,ti OR foetal:ab,ti OR foetus:ab,ti OR fetus:ab,ti) AND death:ab,ti) OR ((perinatal:ab,ti OR neonatal:ab,ti) AND (mortality:ab,ti OR morbidity:ab,ti)) OR (diagnostic AND zone*:ab,ti) OR 'lipocalin 2':ab,ti

648426 records

#39 #37 OR #38

1797424 records

Combination search RQ1A (GDM AND phenotype/biomarkers/age-ethnicity-social/lifestyle factors AND stratification/risk AND perinatal outcomes)

#40 #3 AND #33 AND #36 AND #39

14535 records

AND [embase]/lim NOT ([embase]/lim AND [medline]/lim)

6388 records

Filter Human, English

5600 records

NOT 'conference abstract':it

1705 records

NOT ‘review’:it

1319 records

Q1
PubMED
#1
"Diabetes, Gestational"[Mesh]
=15181

#2
"gestational diabetes"[Title/Abstract] OR GDM[Title/Abstract] OR CGM[Title/Abstract] OR pregnancy induced diabetes[Title/Abstract] OR pregnancy-induced diabetes[Title/Abstract]
=21947

#3
#1 OR #2
=25718

2022-03-04
1A
BMI, Biomarkers, age, ethnicity, sociocultural factors, behavioral factors, diet, exercise

#4
((((((((((((((("Phenotype"[Mesh]) OR "Genetic Heterogeneity"[Mesh]) OR "Insulin Resistance"[Mesh]) OR "Genomics"[Mesh]) OR "Metabolomics"[Mesh]) OR "Proteomics"[Mesh]) OR "Lipidomics"[Mesh]) OR "MicroRNAs"[Mesh]) OR "Lipids"[Mesh]) OR "Triglycerides"[Mesh]) OR "Adipokines"[Mesh]) OR "Leptin"[Mesh]) OR "Adiponectin"[Mesh]) OR "Pregnancy-Associated Plasma Protein-A"[Mesh] OR "biomarkers"[Mesh]) OR "exosomes"[Mesh])

=2554133

#5

Sex hormone-binding globulin[Title/Abstract] OR SHBG[Title/Abstract] OR estrogen[Title/Abstract] OR cortisol[Title/Abstract] OR prolactin[Title/Abstract] OR progesterone[Title/Abstract] OR insulin-like growth factor[Title/Abstract] OR IGF[Title/Abstract] OR placenta-derived[Title/Abstract] OR human placental lactogen[Title/Abstract] OR hPL[Title/Abstract] OR human chorionic somatomammotropin[Title/Abstract] OR HCS pregnancy associated plasma protein A[Title/Abstract] OR PAPP-A[Title/Abstract] OR Placental growth hormone[Title/Abstract] OR PGH[Title/Abstract] OR Thyroid*[Title/Abstract] OR TSH[Title/Abstract] OR thyroglobulin antibody[Title/Abstract] OR thyroid peroxidase antibody[Title/Abstract] OR "free T4"[Title/Abstract] OR "free T3"[Title/Abstract] OR Metabolomics*[Title/Abstract] OR butyrate[Title/Abstract] OR butyric acid[Title/Abstract] OR acetylcarnitine[Title/Abstract] OR acylcarnitine[Title/Abstract] OR carnitine[Title/Abstract] OR lactate[Title/Abstract] OR lactic acid[Title/Abstract] OR Genome-Wide Association Study[Title/Abstract] OR GWAS[Title/Abstract] OR single nucleotide variation[Title/Abstract] OR Polymorphism, Single Nucleotide[Title/Abstract] OR SNP[Title/Abstract] OR genetic variation[Title/Abstract] OR genetic risk score[Title/Abstract] OR genotype risk score[Title/Abstract] OR GRS[Title/Abstract] OR polygenic risk score[Title/Abstract] OR polygenic score[Title/Abstract] OR PRS[Title/Abstract] OR CpG Islands[Title/Abstract] OR DNA Methylation[Title/Abstract] OR Epigenomes[Title/Abstract] OR Methylome[Title/Abstract]

=950352

#6

lipids OR triacylglycerol*[Title/Abstract] OR triglyceride*[Title/Abstract] OR VLDL[Title/Abstract] OR very low density lipoprotein[Title/Abstract] OR cholesterol OR lipoprotein* OR hdl[Title/Abstract] OR ldl[Title/Abstract] OR hyperlipidemia* OR apolipoprotein*[Title/Abstract] OR non-hdl[Title/Abstract] OR lipidemia*[Title/Abstract] OR lipemia*[Title/Abstract] OR Lipemic[Title/Abstract] OR dyslipidemia OR fatty acid[Title/Abstract] OR Amino Acid* OR aminoacid* OR bcaa[Title/Abstract] OR creatine[Title/Abstract] OR leucine[Title/Abstract] OR isoleucine[Title/Abstract] OR valine[Title/Abstract] OR alanine[Title/Abstract] OR glutamine[Title/Abstract] OR glutamate[Title/Abstract] OR betaine[Title/Abstract] OR creatine [Title/Abstract] OR aspartate[Title/Abstract] OR tyrosine[Title/Abstract] OR

glutathione[Title/Abstract] OR serine[Title/Abstract] OR threonine[Title/Abstract] OR histidine[Title/Abstract] OR tryptophan[Title/Abstract] OR glycine[Title/Abstract] OR inflammation OR Cytokine*[Title/Abstract] OR interluekin*[Title/Abstract] OR IL[Title/Abstract] OR CRP[Title/Abstract] OR high-sensitivity CRP[Title/Abstract] OR hs-CRP[Title/Abstract] OR TNF-alpha OR Tumor Necrosis Factor alpha OR MCP-1 OR Monocyte Chemoattractant Protein-1 OR chemokine OR adipokines[Title/Abstract] OR adipocytokines[Title/Abstract] OR adiponectin[Title/Abstract] OR leptin[Title/Abstract] OR RBP4[Title/Abstract] OR retinol binding protein 4[Title/Abstract] OR exosome*[Title/Abstract]=4363686

#7
#4 OR #5 OR #6
=5991911

#8
age[Title/Abstract] OR ethnic*[Title/Abstract] OR white[Title/Abstract] OR caucasian[Title/Abstract] OR asian[Title/Abstract] OR african[Title/Abstract] OR american[Title/Abstract]
=3322327

#9
"Exercise"[Mesh] OR exercise[Title/Abstract] OR physical activit*[Title/Abstract]
=484766

#10
BMI[Title/Abstract] OR body mass[Title/Abstract] OR bodymass[Title/Abstract] OR "body mass index"[Title/Abstract] OR body composition[Title/Abstract] OR anthropometr*[Title/Abstract] OR obes*[Title/Abstract] OR overweight[Title/Abstract] OR "over weight"[Title/Abstract] OR excess body weight[Title/Abstract] OR excess body fat[Title/Abstract] OR excess weight[Title/Abstract] OR body weight[Title/Abstract] OR bodyweight[Title/Abstract] OR lean[Title/Abstract] OR fat[Title/Abstract] OR muscle mass[Title/Abstract] OR musclemass[Title/Abstract]
=1001815

#11
(("Diet Therapy"[Mesh]) OR "Diet"[Mesh]) OR ((diet[Title/Abstract] OR food[Title/Abstract] OR nutriti*[Title/Abstract] OR dietary[Title/Abstract] OR "eating habit*"[Title/Abstract]) AND (composition*[Title/Abstract] OR pattern*[Title/Abstract] OR matrix*[Title/Abstract] OR "percent calories"[Title/Abstract]))
=433783

#12
(“Health behavior"[Mesh]) OR ((behavio*[Title/Abstract]) AND (health*[Title/Abstract]))
=589285

#13
#7 OR #8 OR #9 OR #10 OR #11 OR #12
=9842933

Subclassification

#14
subtype*[Title/Abstract] OR subclassif*[Title/Abstract] OR stratif*[Title/Abstract] OR subgroup*[Title/Abstract]
=694723

Risk/Prediction

#15
prognos*[Title/Abstract] OR progress*[Title/Abstract] OR predict*[Title/Abstract] OR model*[Title/Abstract] OR nomogram*[Title/Abstract] OR statistical[Title/Abstract] OR score*[Title/Abstract] OR risk categor*[Title/Abstract] OR risk

factor*[Title/Abstract] OR risk assessment[Title/Abstract] OR algorithm*[Title/Abstract] OR equation*[Title/Abstract] OR precision[Title/Abstract] OR personali*[Title/Abstract]
=7803055

#16
#13 OR #14
=8128024

Perinatal outcomes

#17
Perinatal outcome*[Title/Abstract] OR pregnancy outcome*[Title/Abstract] OR "large for gestational age"[Title/Abstract] OR LGA[Title/Abstract] OR birthweight[Title/Abstract] OR small-for-gestational-age SGA preterm*[Title/Abstract] OR "cesarean deliver*"[Title/Abstract] OR large-for-gestational-age[Title/Abstract] OR neonatal respiratory morbidity[Title/Abstract] OR neonatal respiratory stress[Title/Abstract] OR hypoglycemia[Title/Abstract] OR hypoglycaemia[Title/Abstract] OR polycythemia[Title/Abstract] OR macrosomi*[Title/Abstract] OR "birth injur*"[Title/Abstract] OR hyperbilirubinemia[Title/Abstract] OR congenital malformations[Title/Abstract] OR intrauterine death[Title/Abstract] OR fetal outcome*[Title/Abstract] OR perinatal mortality[Title/Abstract] OR perinatal morbidit*[Title/Abstract]
=159004

#18
((("Hypoglycemia"[Mesh]) OR "Polycythemia"[Mesh]) OR "Respiratory Distress Syndrome, Newborn"[Mesh]) OR "Perinatal Death"[Mesh]
=53188

#19
#17 OR #18
=186624

Combined

#20
#3 AND #13 AND #16 AND #19
=3497

#21
#20 Filters: Humans, English
=**2861 references**

#22
(review[Title/Abstract]) OR (review[Publication Type])
=3665456

#23
#21 NOT #22
=**2442 references**

Supplementary Note 2: Modified Joanna Briggs Institute critical appraisal checklist for cohort studies

Modified JBI Critical Appraisal Checklist

This form captures several categories of factors that could induce bias 1) selection, 2) confounding, 3) the outcome was not present at the time of exposure assessment (eg, time of precision variable assessment), 4) loss to follow-up

Selection

The following questions are used to assess whether the method of selecting GDM cases, GDM subtypes, or GDM precisions variables may have resulted in biased findings or conclusions. If there are potential biases, then consider carefully whether the study should be included.

1a) Sample size.

Are there enough GDM cases in each subtype, or with the precision variable measured, for us to draw reliable conclusions. For instance, potentially 30-50 in each GDM group?

Check all that apply.

O Yes O No O Unclear O Not applicable

1b) Optional: supporting text or note

2a) Are both subtypes selected from the same community/population; or if the subtype is sociocultural, are the subtypes selected with the same criteria. (This is unlikely to occur frequently)

Check all that apply.

O Yes O No O Unclear O Not applicable

2b) Optional: supporting text or notes

3a) Were the subtypes measured similarly in all women.

Highest quality is if the same method is used for all participants.

Check all that apply.

O Yes O No O Unclear O Not applicable

3b) Optional: supporting text or notes

4a) Were the subgroups ascertained in a reliable way (eg, hospital record, interview).

Secure record or structured interview are considered highest quality. Written self-report or medical record are considered mediocre quality. No description is poor quality.

Check all that apply.

O Yes O No O Unclear O Not applicable

4b) Optional: supporting text or notes

Confounding

The purpose of these questions is to help guide us to <u>think about whether we can draw valid conclusions</u> from the study, or whether other factors (confounders) could be influencing the findings.

5a) Were confounding factors identified.

a confounder is a variable that influences both the exposure variable and outcome variable, that isn't on the causal pathway and results in a spurious association

Check all that apply.

O Yes O No O Unclear O Not applicable

5b) Optional: supporting text or notes

6a) Were strategies to deal with confounding stated.

Adjusted analyses, or subgroup analyses could count. For instance, the study controls for BMI (or other potential confounders) when comparing outcomes between subtypes

Check all that apply.

O Yes O No O Unclear O Not applicable

6b) Optional: supporting text or notes

Outcome

The purpose of this section is to assess whether the individuals had the outcome when the subtype was measured. We are trying to assess/establish whether temporality between exposure and outcome is well defined.

7a) Were the groups free of the outcome at the time of subtype measurement.

The goal of this question is to assess whether the subtype or precision variable influences the outcome. Said differently, the goal is to assess temporality of exposure and outcome (even though we understand that sometimes the pathophysiology of the outcomes we are interested in begin in early pregnancy).

Check all that apply.

O Yes O No O Unclear O Not applicable

7b) Optional: supporting text or note

8a) Were the outcomes measured in a valid and reliable way.

If there are multiple outcomes and not all are measured well, please make a note. Overall, the goal is to help you determine whether the study should be included.

Check all that apply.

O Yes O No O Unclear O Not applicable

8b) Optional: supporting text or notes

Loss to follow-up

If there was substantial loss to follow-up (eg, >25%), then are the women lost inherently different and could this result in systematic differences in outcomes. The goal is to help us think about whether the loss to follow-up results in a lower quality study that we would be unable to draw valid conclusions from.

9a) Was there adequate follow up of all participants.

The goal is to assess whether any bias could be introduced due to loss to follow-up. High quality is complete follow up (all accounted for), or the % lost to follow up is unlikely to introduce bias, or there was a description of those lost.

Check all that apply.

O Yes O No O Unclear O Not applicable

9b) Optional: supporting text or notes.

10a) Was appropriate statistical analysis used.

Check all that apply.

O Yes O No O Unclear O Not applicable

10b) Optional: supporting text or notes.

11a) Overall appraisal.

Please pick exclude if we should not summarize OR meta analyze the study.

Check all that apply.

O Yes O No

12a) Overall quality.

Check all that apply.

O High O Low

11a-12a) Optional: supporting text or notes of overall appraisal.

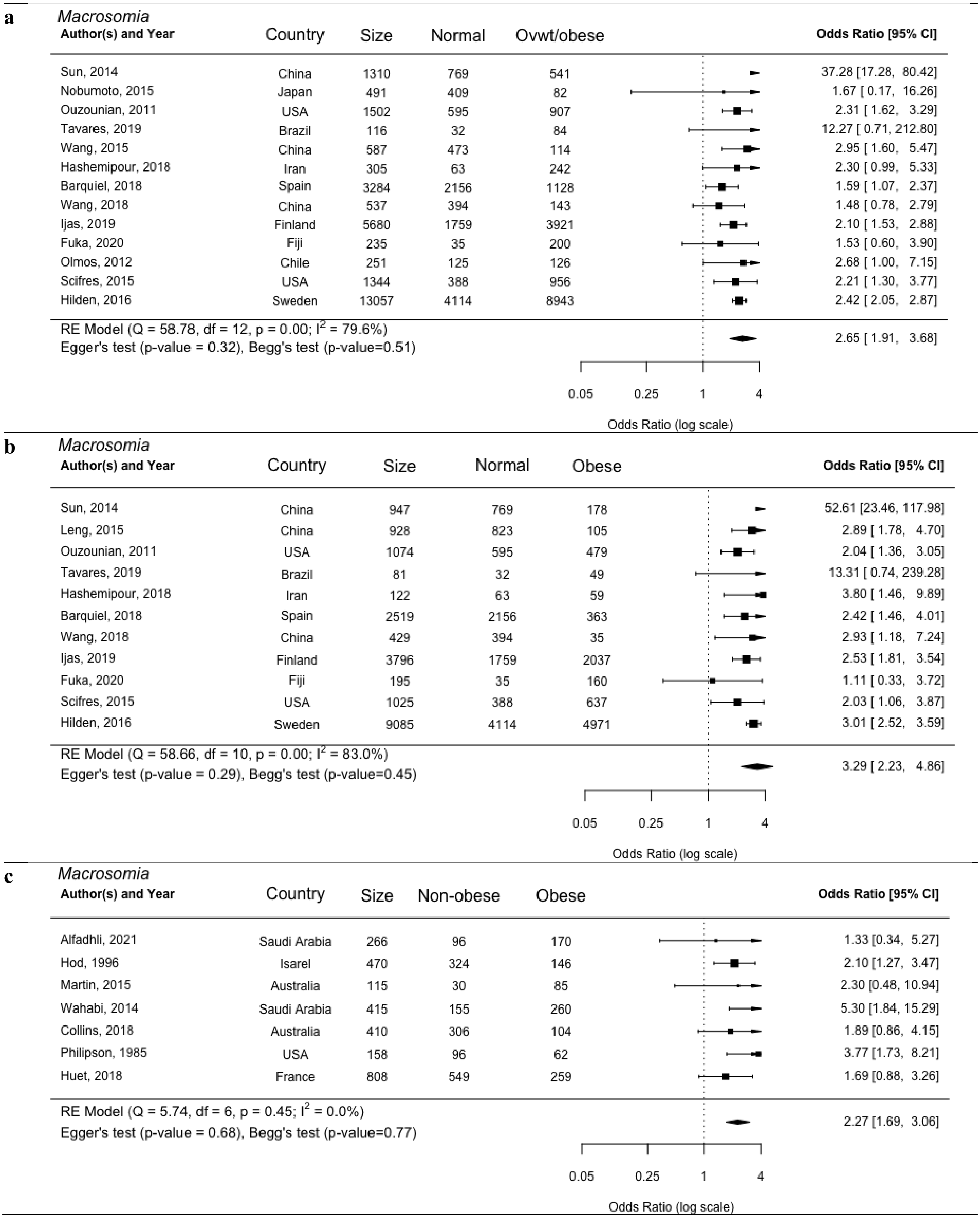

Supplementary Figure 1: Summary odds ratio (95% CI) of macrosomia for maternal body mass index categories

Panel A: maternal BMI categorized as overweight vs. BMI normal range and offspring macrosomia.

Panel B: maternal BMI categorized as obesity vs. BMI normal range and offspring macrosomia.

Panel C: maternal BMI categorized as obesity vs. BMI not obese and offspring macrosomia.

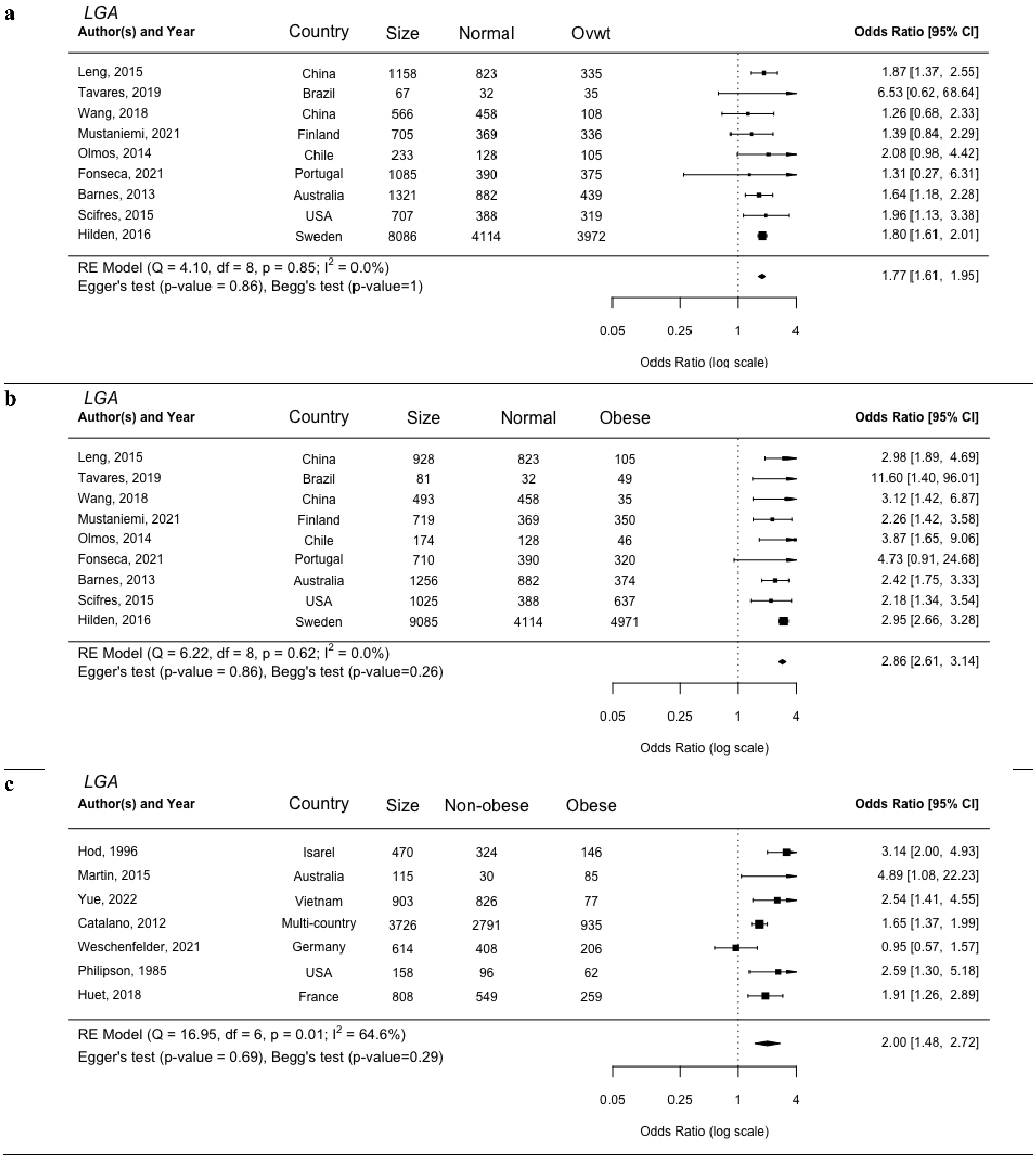

Supplementary Figure 2: Summary odds ratio (95% CI) of large-for-gestational age (LGA) for maternal body mass index categories

Panel a: maternal BMI categorized as overweight vs. BMI normal range and offspring LGA.
Panel b: maternal BMI categorized as obesity vs. BMI normal range and offspring LGA.
Panel c: maternal BMI categorized as obesity vs. BMI not obese and offspring LGA.
Abbreviations: LGA: large-for-gestational age

Supplementary Table 1: Subgroup meta-analyses of overweight/obese vs. normal BMI, and continuous BMI: per unit increase and association with macrosomia.

**Subgroup**	**Number of studies**	**Pooled RR (95% CI)**
Overweight/obese BMI	13	
Study year		
Prior to 2010	9	2.73 (1.83, 4.06)
2010 or later	4	2.48 (1.61, 3.83)
Study quality		
low	3	6.44 (0.84, 49.42)
high	10	2.21 (1.96, 2.49)
Adjusted for covariates *		
Yes	0	NA
Continuous BMI	3	
Study year		
Prior to 2010	1	1.17 (1.12, 1.22)
2010 or later	2	1.08 (1.02, 1.15)
Study quality		
low	0	NA
high	3	1.12 (1.05, 1.19)
Adjusted for covariates		
Yes	2	1.16 (1.11, 1.20)

*No studies were adjusted for covariates. All studies reported <25, 25-30, 30+, thus ORs of overweight/obesity vs. normal from individual studies were derived from 2x2 table by pooling outcome data of BMI 25-30 and 30+ groups

Supplementary Table 2: Subgroup meta-analyses of overweight/obese vs. normal BMI, and continuous BMI: per unit increase and association with large-for gestational age.

Subgroup	Number of studies	Pooled RR (95% CI)
Overweight/obese BMI	10	
Study year		
Prior to 2010	6	2.32 (2.13, 2.53)
2010 or later	4	2.03 (1.34, 3.06)
Study quality		
low	2	2.02 (1.41, 2.89)
high	8	2.21 (1.92, 2.54)
Adjusted for covariates *		
Yes	0	NA
Continuous BMI	10	
Study year		
Prior to 2010	6	1.11 (1.07, 1.16)
2010 or later	4	1.05 (1.02, 1.08)
Study quality		
low	0	NA
high	10	1.09 (1.06, 1.12)
Adjusted for covariates		
Yes	5	1.11 (1.06, 1.15)

*No studies were adjusted for covariates. All studies reported <25, 25-30, 30+, thus ORs of overweight/obesity vs. normal from individual studies were derived from 2x2 table by pooling outcome data of BMI 25-30 and 30+ groups
