# Supplementary material for: Refining the diagnosis of gestational diabetes mellitus: a systematic review and meta-analysis
Source: Commun Med (Lond). 2023 Dec 18;3:185. doi: 10.1038/s43856-023-00393-8 (PMC10728189; doi:10.1038/s43856-023-00393-8)
Supplement: Supplementary file 8 — Reporting Summary [file 43856_2023_393_MOESM8_ESM.pdf]

nature portfolio

COMMSMED-23-0362
Corresponding author(s): Ellen C. Francis, PhD
Last updated by author(s): Oct 2, 2023

Reporting Summary

Nature Portfolio wishes to improve the reproducibility of the work that we publish. This form provides structure for consistency and transparency in reporting. For further information on Nature Portfolio policies, see our Editorial Policies and the Editorial Policy Checklist.

Statistics

For all statistical analyses, confirm that the following items are present in the figure legend, table legend, main text, or Methods section.

n/a	Confirmed	
☒	☐	The exact sample size (*n*) for each experimental group/condition, given as a discrete number and unit of measurement
☒	☐	A statement on whether measurements were taken from distinct samples or whether the same sample was measured repeatedly
☐	☒	The statistical test(s) used AND whether they are one- or two-sided<br>*Only common tests should be described solely by name; describe more complex techniques in the Methods section.*
☒	☐	A description of all covariates tested
☐	☒	A description of any assumptions or corrections, such as tests of normality and adjustment for multiple comparisons
☐	☒	A full description of the statistical parameters including central tendency (e.g. means) or other basic estimates (e.g. regression coefficient) AND variation (e.g. standard deviation) or associated estimates of uncertainty (e.g. confidence intervals)
☐	☒	For null hypothesis testing, the test statistic (e.g. *F*, *t*, *r*) with confidence intervals, effect sizes, degrees of freedom and *P* value noted<br>*Give P values as exact values whenever suitable.*
☒	☐	For Bayesian analysis, information on the choice of priors and Markov chain Monte Carlo settings
☒	☐	For hierarchical and complex designs, identification of the appropriate level for tests and full reporting of outcomes
☐	☒	Estimates of effect sizes (e.g. Cohen's *d*, Pearson's *r*), indicating how they were calculated

Our web collection on statistics for biologists contains articles on many of the points above.

Software and code

Policy information about availability of computer code

Data collection	NA
Data analysis	Meta-analyses were conducted using R (version 4.2.3) and the 'metafor' R package (version 4.0-0).

For manuscripts utilizing custom algorithms or software that are central to the research but not yet described in published literature, software must be made available to editors and reviewers. We strongly encourage code deposition in a community repository (e.g. GitHub). See the Nature Portfolio guidelines for submitting code & software for further information.

Data

Policy information about availability of data

All manuscripts must include a data availability statement. This statement should provide the following information, where applicable:

- Accession codes, unique identifiers, or web links for publicly available datasets
- A description of any restrictions on data availability
- For clinical datasets or third party data, please ensure that the statement adheres to our policy

Data are available upon reasonable request

Research involving human participants, their data, or biological material

Policy information about studies with human participants or human data. See also policy information about sex, gender (identity/presentation), and sexual orientation and race, ethnicity and racism.

Reporting on sex and gender	The focus of this systematic review was on pregnant individuals/women only
Reporting on race, ethnicity, or other socially relevant groupings	In this systematic review we examined race/ethnicity as it was originally reported in the primary studies
Population characteristics	NA
Recruitment	NA
Ethics oversight	NA

Note that full information on the approval of the study protocol must also be provided in the manuscript.

Field-specific reporting

Please select the one below that is the best fit for your research. If you are not sure, read the appropriate sections before making your selection.

☐ Life sciences ☒ Behavioural & social sciences ☐ Ecological, evolutionary & environmental sciences

For a reference copy of the document with all sections, see nature.com/documents/nr-reporting-summary-flat.pdf

Behavioural & social sciences study design

All studies must disclose on these points even when the disclosure is negative.

Study description	A systematic review that included qualitative summaries and quantitative meta analysis
Research sample	Systematic literature searches were performed in PubMed (https://pubmed.ncbi.nlm.nih.gov/) and EMBASE (https://www.embase.com)
Sampling strategy	Search terms and strategy are included in supplementary material 1
Data collection	All studies were screened by at least two reviewers and conflicts were resolved by a third independent reviewer. All titles and abstracts were screened for eligibility, and those that were assessed as potentially meeting inclusion/exclusion criteria were selected for full-text evaluation. During full-text evaluation, if the study referenced potentially relevant studies, we included those in full-text evaluation
Timing	from database inception to March 2022.
Data exclusions	As our main goal was to review studies identifying GDM sub-phenotypes beyond glycemia, we excluded studies that only reported on glycemic markers or thresholds (e.g., HbA1c, fasting glucose, oral glucose tolerance test [OGTT] glycemic values), or studies that were focused on assessing differences in outcomes based on timing of glucose measurement.<br>We also excluded studies that 1) measured the precision marker after GDM diagnosis (e.g., total gestational weight gain [GWG] over the whole pregnancy, or fetal biometry after 32 weeks' gestation), 2) combined pre-existing diabetes or overt diabetes (based on non-pregnancy glycemic thresholds) with GDM, 3) included women with multi-gestations, or 4) did not contain full-length manuscripts in English.
Non-participation	NA
Randomization	NA

Reporting for specific materials, systems and methods

We require information from authors about some types of materials, experimental systems and methods used in many studies. Here, indicate whether each material, system or method listed is relevant to your study. If you are not sure if a list item applies to your research, read the appropriate section before selecting a response.

Materials & experimental systems

n/a	Involved in the study
☒	☐ Antibodies
☒	☐ Eukaryotic cell lines
☒	☐ Palaeontology and archaeology
☒	☐ Animals and other organisms
☐	☒ Clinical data
☒	☐ Dual use research of concern
☒	☐ Plants

Methods

n/a	Involved in the study
☒	☐ ChIP-seq
☒	☐ Flow cytometry
☒	☐ MRI-based neuroimaging

Clinical data

Policy information about clinical studies

All manuscripts should comply with the ICMJE guidelines for publication of clinical research and a completed CONSORT checklist must be included with all submissions.

Clinical trial registration	NA
Study protocol	A protocol for this review was registered at PROSPERO (CRD42022316260) on 11 March 2022.
Data collection	data were abstracted from full-texts that were published prior to March 2022
Outcomes	maternal hypertensive disorders in pregnancy, cesarean delivery, offspring anthropometry at birth (macrosomia, LGA, small-for-gestational-age [SGA]), preterm delivery, birth trauma, metabolic sequelae (e.g., hypoglycemia) or mortality
